# Vitamin D in early life and later risk of multiple sclerosis—A systematic review, meta-analysis

**DOI:** 10.1371/journal.pone.0221645

**Published:** 2019-08-27

**Authors:** Kamila Ismailova, Pratiksha Poudel, Alexandr Parlesak, Peder Frederiksen, Berit L. Heitmann

**Affiliations:** 1 Research Unit for Dietary Studies, The Parker Institute, Bispebjerg and Frederiksberg Hospital, Copenhagen, Denmark; 2 School of Global Health, University of Copenhagen, Copenhagen, Denmark; 3 University College Copenhagen, Copenhagen, Denmark; 4 The Boden Institute of Obesity, Nutrition, Exercise, and Eating Disorders, The University of Sydney, Sydney, Australia; 5 Section for General Practice, Department of Public Health, University of Copenhagen, Copenhagen, Denmark; University of Missouri Columbia, UNITED STATES

## Abstract

The study examined results from previous studies of early life vitamin D exposure and risk of MS in adulthood, including studies on season or month of birth and of migration. A systematic review was conducted using PubMed and Web of Science databases as well as checking references cited in articles. The quality of studies was assessed using the Newcastle-Ottawa scale and the AMSTAR score. Twenty-eight studies were selected for analysis. Of these, six population studies investigated early life vitamin D exposure and risk of MS, and three found inverse while the remaining found no associations. A consistent seasonal tendency for MS seemed evident from 11/15 studies, finding a reduced occurrence of MS for Northern hemisphere children who were born late autumn, and late fall for children born in the Southern hemisphere. This was also confirmed by pooled analysis of 6/15 studies. Results of the migration studies showed an increased risk of MS if migration from high to low-MS-risk areas had occurred after age 15 years, while risk of MS was reduced for those migrating earlier in life (<15years). A similar, but inverse risk pattern was observed among migrants from low to high-MS-risk areas. One study found an increased risk of MS in the second generation of migrants when migrating from low to high-MS-risk areas. An association between early life vitamin D and later risk of MS seems possible, however evidence is still insufficient to conclude that low vitamin D exposure in early life increases the risk of MS in adulthood.

PROSPERO register number: CRD 42016043229.

## Introduction

Multiple sclerosis (MS) is a chronic autoimmune demyelinating disease that affects the Central Nervous System (CNS). It is one of the most common neurological disorders occurring in young and middle-aged adults and is more common in women than men [1,2]. It is affecting more than 2.1 million people worldwide and its prevalence is increasing [1]. Even though some patients with MS may experience a light form of disability only, more than 60% of those with MS are no longer capable of working. In most cases, the disease decreases the quality of patients’s life and causes a considerable health and economic burden to society [3]. It is, therefore, vital to search for aetiological factors for MS and to identify cost-effective preventive strategies to reduce the burden of disease.

The aetiology of MS is currently unknown although assumed to be caused by a combination of genetic and environmental factors [4]. However, the uneven geographic distribution of the MS is apparent from epidemiological studies where increased rates are seen in high-latitude regions, while MS is uncommon close to the equator, leading to north-south prevalence gradient [5]. The MS distribution was categorized by Kurtzke et al. [6] according to MS prevalence rates in three geographical zones: high, medium and low prevalence areas. This unequal distribution is expected to be amongst others due to higher vitamin D levels via sun exposure in warmer climates (low risk regions), while lower in cooler climates (high risk regions).

An increasing body of evidence suggests that the disease may be associated with exposure to the Epstein-Barr virus, smoking and high body mass index in childhood and adolescence [7–10]. Moreover, in recent years, vitamin D insufficiency has been suggested as potential predisposing factors for development of MS [1,11,12]. In this regard, it has been hypothesized that a higher availability of vitamin D may reduce the immune system’s inflammatory response and hence reduce the risk of autoimmune disorders leading to MS [13]. Furthermore, recent research suggests that low vitamin D occurring during sensitive stages of development, such pre- and perinatal life may be a particular concern in relation to later risk of MS [14]. Experiments on rats, using developmental vitamin D deficiency models, have shown alterations in the brain of offspring from vitamin D deficient compared to vitamin D sufficient rat mothers, including a reduced cell differentiation, and expression of neurotrophic factors, effects that may persist into adulthood [15]. However, results from studies on experimental autoimmune encephalomyelitis from animal models, on the role of vitamin D during early development are limited and inconsistent [16]. So far, results from human studies that examined the influence of human vitamin D availability in early life (pre- and perinatal period) on the risk of MS in adulthood have not been comprehensively summarized, and we therefore conducted a systematic literature review of the results from available intervention and population studies. As season, latitude and migration relates to sun and UVB exposure, and may provide correlational type of evidence relating early life vitamin D exposure to MS, this review also includes a meta-analysis of pooled results from season or month of birth, and an update of studies examining latitudinal gradients and age and generation at migration studies in relation to later MS occurrence.

## Methods

### Protocol and registration

Search methods have been explicitly defined in advance in order to reduce researcher bias (PROSPERO 2016: CRD 42016043229).

### Search methods and terms used

The literature search was performed through the PubMed/Medline platform in July 2018. Additionally, Web of Science was used to identify articles cited in included publications after having checked the reference list of retrieved papers. The terms used for the article search combined key terms for vitamin D, month or season of birth, migration and multiple sclerosis (for further details, see S1 Table). The search using the same search strategy was updated before the final analysis to retrieve further studies for inclusion.

### Selection criteria

Two independent researchers performed the selection of papers and examined titles and abstracts to exclude studies that did not meet predefined inclusion criteria. Then, full papers were screened and selected for this review. In case of uncertainty, a third researcher was involved. The PRISMA Statement was used as a guideline [17].

### Eligibility criteria for inclusion in this review

Predefined inclusion criteria stated that studies must:

i) Investigate the association between early life vitamin D and the risk of developing MS in adulthood. The review limited itself to the pre- (the time between conception and birth) and perinatal period (from birth to 1 month); ii) Assess the season or month of birth as risk factor for development of MS; iii) Examine the difference in risk of MS between early and late migrants or between the first and the second generation of migrants. The region where the studies were conducted is important in order to understand possible relationships between the season or the month-of-birth effects on MS susceptibility for different latitudinal gradients [18]. Therefore, there was no limitation on study location.

### Types of studies

To be included in this review articles had to adhere to one of the following study designs: (i) intervention studies, (ii) cohort studies, case-control studies; nested case-control studies, case-cohort studies, or cross-sectional studies; (iii) systematic reviews.

### Characteristics of participants

To be included in this review, publications were included only if they comprised participants that met the following criteria: (i) for vitamin D analysis: pregnant women without MS and neonates (from birth to 1 month); (ii) for season/month of birth and migration analysis: adult patients with MS (diagnosed by any criterion) and affected by any type of MS such as relapsing remitting, secondary progressive, primary progressive, progressive relapsing.

### Types of interventions/exposures

Studies were selected for inclusion based on the following types of exposures: (i): vitamin D (ii) season/month of birth; (iii) migration (age at migration, changes in latitude regions, and the risk difference between generations of migrants).

### Types of outcome measures

The outcome of interest was: diagnosis of MS / adults patients with MS

### Exclusion criteria

The following types of publications were not included: (i) papers not published in English; (ii) case reports, case series, study protocols, opinion documentations, letters, editorials or commentaries, dissertations, book reviews, common (non-systematic) reviews and animal studies.

### Data extraction and quality assessment

On each included publication, information was extracted and tables created with information on the author, year of publication, study objectives, design, setting, sample size, age, and gender of MS patients, exposure, main findings and methodological quality. For the season or month of birth analysis, additional data was extracted on estimated latitude of country. If the original paper did not include this information Google map was used to determine an estimated mean latitude of the country. At the level of the systematic review, data was extracted on author name, year of publication, number of included studies, methodological quality and main findings, while for migration studies data was extracted on author and year, country of origin, country of migration, main findings and methodological quality. At the level of meta-analysis, numbers of observed and expected births of MS patients for each month and risk estimates were extracted.

The quality of included studies was assessed using the Newcastle-Ottawa scale for observational studies, and the Assessment of Multiple Systematic Reviews (AMSTAR) score for systematic reviews [19]. A quality score for observational studies was calculated based on three major components of the Newcastle-Ottawa scale: (1) selection of the groups included to the study, (2) comparability, (3) assessment of the outcome or exposure. The maximum score was 9 points, representing the highest methodological quality, while a quality score of systematic reviews was categorized as low (0–3), moderate (4–7) or high (8–11) according to the AMSTAR score instructions (for further details, see S3 File).

### Statistical analysis

#### Month of birth and risk of MS

In total 6/15 studies provided full statistical information on month of birth and subsequent MS risk and were included for this meta-analysis. From each study we extracted the observed and expected number of MS in each month. The expected number represent the expected number of MS if there were no seasonal variation by month. Standardized incidence ratios (SIR) were calculated as the ratio between observed and expected MS. The standard error of log (SIR) was calculated as 1 divided by the square root of the observed number O of MS, hence the expected number is treated as a known background incidence of MS in the population. The SIR’s were combined using separate random-effects meta-analyses. Heterogeneity between studies was assessed by the *I*^*2*^ statistics [20]. The analysis was carried out in R version 3.5.1 [21] using the Meta package [22].

## Results

### Study selection

**Fig 1:**
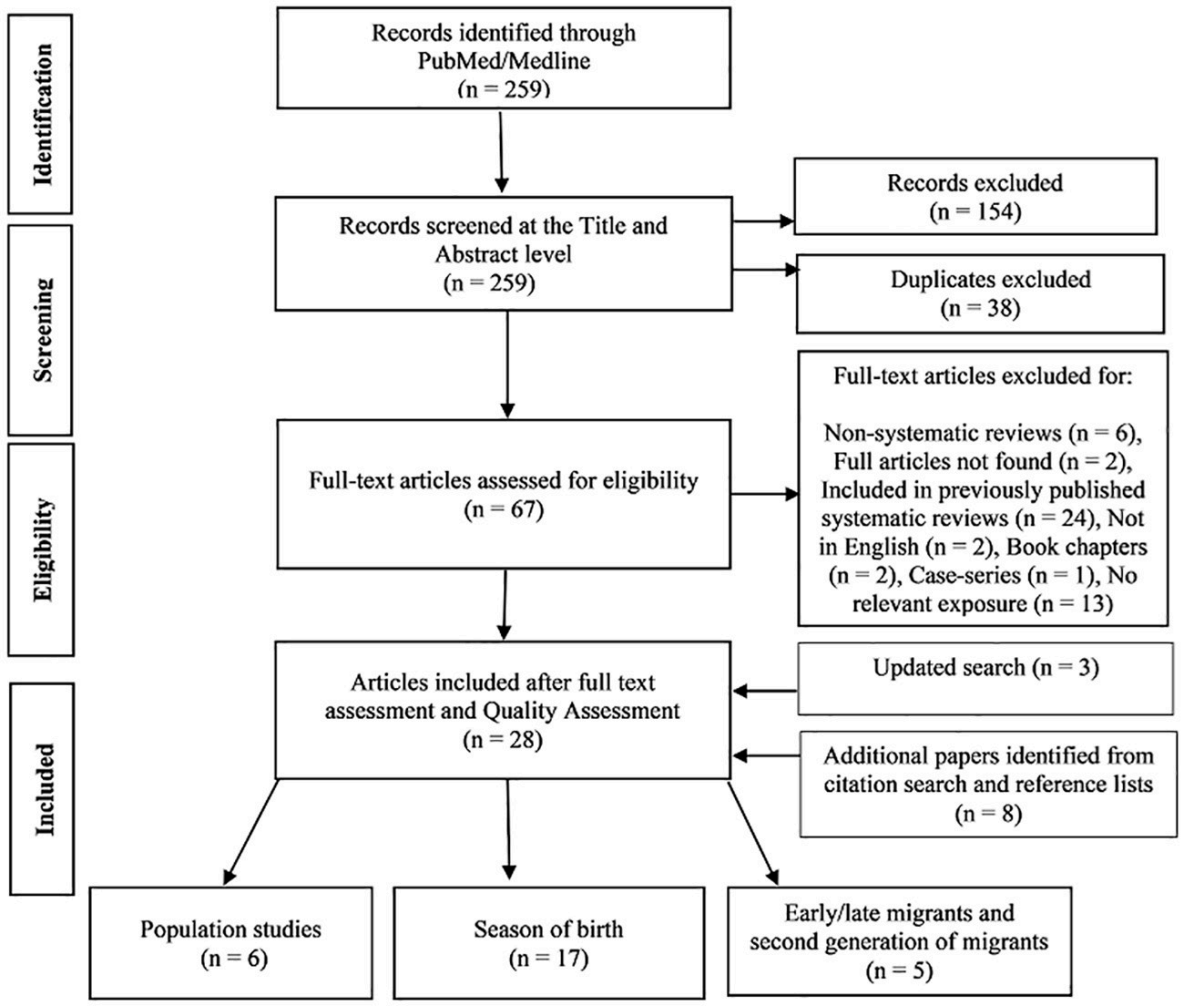
Flow chart with information on the identified and excluded studies.

Based on the search strategy, a total of 267 citations (259 from PubMed/Medline and an additional 8 from citation searches and reference lists) were identified. After reviewing the title and/or abstract of all the citations and removing duplicates, 192 citations were excluded as they did not meet the inclusion criteria. For the remaining 67 studies, the full text was retrieved. In order to avoid duplications only studies with original results were included. Furthermore, for the correlation type studies we only included studies that had not been included in two previously published systematic reviews of season or month of birth, and latitudinal gradients and age and generation at migration studies. The same search strategy was performed in July 2018. This revealed additionally three studies. A final number of 28 articles thus met the inclusion criteria. The number of included and excluded articles with reasons for exclusion is shown in (Fig 1).

### Study characteristics

No published intervention studies, but six observational population studies, seventeen season of birth studies and five migration studies were identified (Fig 1). Tables 1–4 feature the characteristics, main findings and quality of the 28 included studies. The studies were highly diverse with respect to design, methods, and participants included. Therefore, a meta-analysis of the results of the included studies could not be performed for all studies. Due to this heterogeneity, the analysis of study types is done separately. However, it was possible to pool the results of 6/15 seasonal studies.

**Table 1:** Characteristics of population studies investigating influence of vitamin D availability in early life and the risk of MS in adulthood.

First author, y (ref)	Objective	Study design	Study setting	Exposure	Median/Mean age at MS onset	Gender distribution of cases n (%)		Population	Main findings	Quality
						Female	Male			
Mirzaei et al. 2011 [28]	To study the effect of gestational vitamin D on adult onset of MS	Cohort	USA	Dietary vitamin D Fortified milk Predicted serum 25(OH)D	n.a	Female nurses 35,372		n = 35,794 mothers of participants, n = 199 MS cases	High maternal milk (RR 0.62, 95%CI 0.40–0.95), vitamin D intake (RR 0.57, 95% CI 0.35–0.91) and serum (25(OH)D) level (RR 0.59, 95% CI 0.37–0.92) associated with lower risk of developing MS in offspring	8
Salzer et al. 2012 [23]	To examine the association between 25-hydroxyvitamin D levels and the risk of MS in blood samples collected prospectively and during gestation	Prospective nested case—control	Sweden	25(OH)D levels during early pregnancy	Median 21 (13–32)	27 (73)	10 (27)	n = 37 cases, n = 185 controls	MS risk in offspring exposed to low gestational 25(OH)D levels (<75 nmol/L vs. ≥75 nmol/L) not different (OR 1.8, 95% CI 0.53–5.8)	6
Ueda et al. 2014 [24]	To assess the relation between neonatal vitamin D concentration, measured in stored blood samples, and risk of MS	Population based case—control	Sweden	25(OH)D levels in newborns	Mean 25.1 [4.7]	349 (76)	110 (24)	n = 459 cases, n = 663 controls	No association between neonatal 25 -hydroxyvitamin D quintile and risk of MS (OR 1.0, 95% CI 0.68–1.44)	8
Cortese et al. 2015 [27]	To investigate the association between vitamin D3 supplementation at different postnatal ages and MS risk	Case—control	Norway	Cod liver oil supplements	Mean 37.6 [10.2]	667 (70)	286 (30)	n = 953 cases, n = 1,717 controls	Supplementation during early childhood (0-12y) did not influence MS risk (OR 1.01, 95% CI 0.81–1.26)	6
Munger et al. 2016 [25]	To examine whether serum 25-hydroxyvitamin D levels in early pregnancy are associated with risk of MS in offspring	Prospective nested case—control	Finland	Maternal serum 25(OH)D levels	Mean 19.8 [3.2]	163 (84)	30 (16)	n = 176 cases, n = 326 controls	Maternal vitamin D deficiency (25(OH)D levels, 12.02 ng/ml) during early pregnancy was associated with nearly two fold higher risk of MS in the offspring (RR 1.90, 95% CI 1.20–3.01)	8
Nielsen et al. 2017 [26]	To examine direct association between level of neonatal vitamin D and risk of MS	Population based case—control	Denmark	25(OH)D levels in newborns	n.a	354 (68)	167 (32)	n = 521 cases, n = 972 controls	In the quantile based analysis, MS risk was highest among individuals in the lowest quintile (<20.7nmol/L vs ≥48.9 nmol/L) (OR 0.53, 95% CI 0.36–0.78). In the analysis of 25(OH)D as a continuous variable, a 25nmol/L increase reduced the risk of MS by 30% (OR 0.70, 95% CI 0.57–0.84)	8

MS, multiple sclerosis; n.a, not available; RR, relative risk; CI, confidence interval; OR, odds ratio; [SD], standard deviation/(range).

**Table 2:** Characteristics of systematic reviews investigating the season or month of birth as a risk factor for development of MS.

First author, y (ref)	Study design	No. of studies included	Hemispheres	Main findings		Quality
				Excess	Reduced	
Torkildsen et al. 2012 [18]	Systematic review	17	Northern hemisphere	April-May	November-December	Moderate
			Southern hemisphere	November	May	
Dobson et al. 2013 [30]	Systematic review and meta-analysis	10	Northern hemisphere	April^1^ (O:E 1.05, p = 0.05)	October^1^ (O:E 0.95, p = 0.04) November (O:E 0.92, p = 0.01)	Moderate
					November^2^ (O:E 0.93, p = 0.04)	
				April^3^ (O:E 1.08, p = 0.001) May (O:E 1.11, p = 0.007) June (O:E 0.94, p = 0.05)	October^3^ (O:E 0.94, p = 0.006) November (O:E 0.89, p = 0.004)	
				April^4^ (O:E 1.08, p = 0.004) May (O:E 1.09, p = 0.002)	October^4^ (O:E 0.95, p = 0.03) November (O:E 0.90,p = 0.03)	
Gale et al. 1995 [51]^a^	Systematic review	28				Moderate

O:E, observed-expected ratio.

^1^All reported data analysis;

^2^Population-conservative analysis;

^3^Geographically-conservative analysis;

^4^Overall-conservative analysis.

^a^This study reviewed migration studies (for the results refer to main text).

**Table 3:** Characteristics of studies assessing the season or month of birth as a risk factor for development of MS.

First author, y (ref)	Study setting	Estimated latitude	Population	Gender distribution of cases n		Median/Mean age at MS onset	Statistic test	Main findings		Quality
				Male	Female			Excess	Reduced	
*Northern hemisphere*										
Gardener et al. 2009 [38]	USA	37°N	n = 723 MS patients	Female nurses 723		n.a	RR (95%CI)	Spring (RR 1.13, 95%CI, 0.88–1.46) Summer (RR1.05, 95%CI, 0.70–1.59)	Fall (RR 0.93, 95%CI, 0.67–1.28) Winter (reference category 1.0)	7
Streym et al. 2013 [42]	Denmark	55.7°N	n = 103 MS patients	n.a	n.a	n.a	HR (95% CI)	April-September	October-March (HR 0.70 95%CI, 0.47–1.04, p = 0.07)	7
Barros et al. (2013) [32]	Portugal (Northwestern region)	41.08°–41.7° N	n = 421 MS patients, n = population control	128	293	44.55	X^2^ and Hewitt	July-December Hewitt (p > 0.05)	n.s	7
Torkildsen et al. 2014 [33]	Norway	62°N	n = 6,649 MS patients, n = population control, MS unaffected: mothers (n = 5,711), fathers (n = 5,247), siblings (n = 8,956)	n.a	n.a	n.a	X^2^, Fishers exact and Bonferroni	April (p = 0.007) Bonferroni (p = 0.09)	n.s	7
Akhtar et al. 2014 [34]	Kuwait	29.3°N	n = 237 MS patients, n = 997,269 non-national births in general population	n.a	n.a	n.a	X^2^ and Hewitt	September-February Hewitt p = 0.09	n.s	5
Akhtar et al. 2015 [35]			n = 1,035 MS patients, n = population control	358	677	n.a	X^2^ and RR (95%CI), Cosinor	December (RR 1.3, 95%CI, 1.1–1.6, p = 0.003) Cosinor p = 0.007	May (RR 0.76, 95%CI, 0.60–0.95, p = 0.015)	5
Tolou-Ghamari et al. 2015 [40]	Isfahan, Iran	32.6°N	n = 1,283 MS patients	304	979	34.6	Descriptive statistics	April, May, September	November	3
Poorolajal et al. 2015 [41]	Hamadan, Iran	34.7°N	n = 100 MS patients, n = 100 control group with acute infectious disease	20	79	36.1	OR (95%CI)	Autumn (September, October, November) (OR 2.77, 95% CI, 1.22–6.30)	Spring (reference category 1.00)	5
Sidhom et al. 2015 [36]	Tunisia	33.8°N	n = 1,912 MS patients, n = population control	545	1,187	n.a	X^2^ and Hewitt	May-October	November-April	6
								X^2^- n.s Hewitt (p = 0.03)		
Rodriguez Cruz et al. 2016 [45]	United Kingdom	53.1°N	n = 6,372 MS patients, (regional MS Cohort)	n.a	n.a	n.a	OR (95%CI) and Walter and Elwood test	April (Obs/Exp 1.24, 95CI%, 1.10–1.41)	November (Obs/Exp 0.84, 95%CI, 0.76–0.92)	6
			n = 21,138 MS patients n = population control					April (Obs/Exp 1.07, 95%CI, 1.02–1.11)	November (Obs/Exp 0.91, 95%CI, 0.87–0.95)	
Balbuena et al. 2016 [43]	Wales	52.1°N	n = 2,927 MS patients, n = population control	n.a	n.a	n.a	X^2^	April (Obs/Exp 1.21 95%CI 1.08–1.36, p < .001)	n.s	5
Villar-Quiles et al. 2016 [37]	Madrid	40.4°N	n = 1,335MS patients, n = population control	439	896	43.4 ± 11.3	X^2^, Hewitt and Roberson	n.s	November (OR 0.76; p = 0.01), January (OR 0.744; p = 0.007), February (OR 0.692; p = 0.001)	6
*Southern hemisphere*										
Becker et al. (2013) [31]	Brazil (south, southeast, and northeast regions)	14.2°S	n = 2,257 MS patients, n = population control	625	1,632	42.1 ± 12.4	X^2^	October, November, December (significant)	April, May, June (significant)	6
Fragoso et al. 2013 [39]	South Amerika (Argentina, Brazil, Chile and Peru)	0–10°S 11–20°S 21–30°S 31–40°S	n = 1,207 MS patients, n = 1,207 controls	351	856	40.8 ± 12.6	Two-way ANOVA and X^2^	n.s		7
*Southern and northern hemisphere*										
Verheul et al. 2013 [44]	Argentina, Australia, Belgium, Brazil, Canada, Cuba, Denmark, Germany, Israel, Italy, Macedonia, Malta, Spain, The Netherlands, Turkey	42°7N	N = 11,415 MS patients n = live-births from national registries	3,618	7,797	n.a	X^2^	April (Obs/Exp = 1.091, X^2^ = 4.950, p = 0.026) June (Obs/Exp = 1.083, X^2^ = 4.210, p = 0.040)	October (Obs/Exp = 0.919, X^2^ = 4.515, p = 0.034) November (Obs/Exp = 0.918, X^2^ = 4.368, p = 0.037)	5

MS, multiple sclerosis; n.a, not available; RR, relative risk; CI, confidence interval; HR, hazard ratio; X^2^, chi-square test; n.s, not significant results; OR, odds ratio; Obs/Exp, observed expected ratio.

**Table 4:** The risk difference in developing MS between early and late age at migration.

First author, y (ref)	Country of origin	Country of migration	Main findings		Quality
			The risk of MS is ↑(increased)/↓(decreased) if migration has been in the period:		
			<15 years	>15 years	
Migration from high to low risk area					
Hammond et al. 2000 [52]	UK and Ireland	Australia	Reduced risk independent of age		n.a
McLeod et al. 2011 [53]	UK and Ireland	Australia	↓	↑	n.a
Migration from low to high risk area					
Cabre et al. 2005 [54]	Martinique and Guadeloupe	Returned back after immigrating to France	↑	↓	6
Berg-Hansen et. al 2015 [55]	Pakistan	Norway			6

n.a, not available; This study [55] investigated effect of migration among the first and second generation of migrants (for statistical values refer to the text).

#### Population studies

Four case-cohort studies examined levels of 25(OH)D from maternal or neonatal blood [23–26], while a fifth case-control study asked cases about cod liver oil supplementation in early childhood [27]. Finally, one [28] population study related maternal milk intake, maternal dietary vitamin D intake and predicted maternal serum to the subsequent offspring risk of MS in adulthood (Table 1). Overall, of the six population studies, three demonstrated significant inverse [25,26,28], and the remaining three studies no associations between early vitamin D exposure and later risk of MS [23,24,27]. Five of the studies were from Nordic countries and one [28] was from the US. Studies were published between 2011 and 2016. The number of cases in the studies ranged from 37 to 953. The mean age of MS patients at the onset of disease ranged from 19 to 37 years.

The US study investigated gestational vitamin D availability by predicted rather than measured maternal 25(OH)D concentrations and by self-reported dietary vitamin D intake during the entire pregnancy [28], while except for one study, the remaining studies used measured neonatal or maternal 25(OH)D concentrations during early pregnancy. Of these studies, four were rated as very good and one as “only” good quality The final study that asked cases about cod liver oil supplementation in early childhood [27], gained the lowest quality among the six studies, both because exposure was assessed over a quite a long age range (ages 0–12), rather than being restricted to pre- or perinatal life, and because of the use of self-administered questionnaires in the ascertainment of exposure, which may have caused recall bias and impacted the validity of the study findings.

All studies were controlled for sex and age, four of the studies performed further multivariate adjusted analysis, although adjustment for relevant covariates such as ethnicity, latitude and season of births did not substantially affect the results [24–26,28]. Due to the using of subjective measures for exposure (e.g. dietary recall, predicted serum 25(OH)D levels) or inadequate representativeness of cases and/or lack of adjustments for important confounders none of the studies reached maximum quality score. For instance, Mirzaei and colleagues [28] assessed maternal serum 25(OH)D levels from non-pregnant women, and Munger and colleagues [25] had measures only from the first trimester of pregnancy; thus, variation in serum 25(OH)D levels in subsequent trimesters, related to likely variations in diet intake of pregnant women over the pregnancy and according to season [29], cannot be excluded.

#### The season or the month of birth as risk factor for MS

Of the identified seventeen studies, two were systematic reviews, one of which also comprised a meta-analysis [18,30] (Table 2). Both reviews were rated of moderate quality (AMSTAR score 4–7) (Table C in S2 Table). The review by Torkildsen et al. [18] reported that most of the total of 17 included studies found a month-of-birth effect, and they concluded that in the Northern hemisphere the birth month of future MS cases seemed to be more frequent in April and May and less frequent in November and December, while a reverse but otherwise similar pattern was identified for the Southern hemisphere. As mentioned above, almost all studies were from the Northern hemisphere (16/17), and all except three (3/17) found associations between month of birth and MS.

The review by Dobson et al. [30] included a meta-analysis and was based on a total of 10 studies published after the year 2000. The meta-analysis found that fewer MS cases than expected occurred in subjects born in October and November, while more than expected MS cases were born in April in the Northern hemisphere. The review performed three types of analysis. In a first population-conservative analysis, they excluded duplicate populations and retained studies with the highest case numbers, only. This analysis showed that particularly November-born infants had a lower later occurrence of MS. In the second geographically conservative analysis, the effect of UV variation over the year was taken into consideration, and more MS cases than expected were observed for subjects born in April, May and June, while fewer diagnosis were made for subjects born in October and November. However, when analysis was performed only including studies of countries with a latitude of less than 52°N, the association between month of birth and later MS diagnosis disappeared with the exception of a higher occurrence of MS among subjects born in June. At latitudes of greater than 52° there is insufficient UV light for cutaneous vitamin D synthesis during half of the year (October through March). Therefore, a latitude of 52° was chosen above which a significant month of birth effect was expected while less variation was expected among populations living at lower latitudes. Finally, a third overall-conservative analysis was performed with inclusion of all studies. Here, the observed MS incidence was higher than expected for those born in April and May, and lower for those born in October and November.

In the present review, we identified and included an additional fifteen studies that were not included in the two previous reviews. Twelve of the fifteen studies were from the Northern hemisphere, of which six reported results from European countries, one from U.S, four from Asia, and one from Africa. Only two studies reported results from countries located in the Southern hemisphere, while one included a worldwide cohort, Table 3.

The sample sizes of the seventeen studies ranged from 100 to 21,138. Seven of the studies selected MS cases based on the McDonald or Poser criteria [31–37], four based on physician-confirmed medical records [38–41], three based on ICD codes [42,43] and MS based registries [44], while one was based on Association of British Neurologist criteria for use of drugs in MS [45]. All studies except four, compared the birth dates of MS patients with that of the general population, The four studies either did not have a comparison group or compared the birth dates of MS patients with birth dates of non-neurological hospital patients, employees and individuals accompanying patients [39,41,42,46]. The studies were also heterogeneous with respect to the statistical analyses. Some used Hewitt, Edwards’ or Cosinor tests while others used Chi-square, and some studies even reported different results depending on which test was used [33,36]. Nevertheless, a consistent seasonal tendency seemed apparent in almost all studies with a generally increased number of MS births in late spring and a reduced one in late autumn, except for four studies that did not demonstrate a significant relation between season or the month of birth and later MS diagnosis [32,34,38,39].

Five studies reported results that were controlled for potential confounders such as place and year of birth [32,38,39,45,47] and two of them indicated a significant month-of-birth association with later MS occurrence [45,47]. Two studies examined seasonality matched for year and region of births [39,45] and one found significant month-of-birth association with later MS diagnosis [45]. The difference in latitudinal gradient of the two studies may explain the variation of their results with one being closer and the other farther from the earth equator. In addition, one study compared births rates of MS patients with fathers (Fisher’s exact test, p = 0.011), mothers (Fisher’s exact test, p = 0.004) or siblings of MS patients free from disease (Fisher’s exact test, p = 0.011), and found a higher incidence among April born MS patients compared to their relatives [33].

In this review, it was furthermore possible to pool the estimates of six studies from Northern hemisphere [43,47–50] in a meta-analysis. The analysis revealed 6% fewer than expected MS cases in March (SIR 0.94, 95%CI, 0.89–1.0) and 12% more MS births than expected in April (SIR 1.12, 95%CI, 1.06–1.18) with moderate heterogeneity in May, June, July and high heterogeneity in November and December (for further details of meta-analysis results by months (S1 Fig)).

The majority of included studies were of comparatively good quality, varying from five to seven AMSTAR points (Tables A and B in S2 Table), while one study was of poor quality due to its descriptive nature of the statistical analyses [40].

The latitudinal gradient of the examined countries ranged from 10°S in the South to 62°N in the North. The limited number of studies from the Southern hemisphere did not allow identification of whether the month of birth patterns in MS differed according to different latitudinal gradients. However, month-of-birth effects were apparent for the countries located at higher latitudes [33,37,43,45] as opposed to the countries located at lower latitudes, and hence being closer to the earth equator [34,39].

#### MS differences in early or late migrants and in the second generation of migrants

One previous systematic review summarized results from migrant studies in relation to risk of MS and differences seen according to time of migration (early and late in life migrants) and among generations of migrants [51]. This review, rated of moderate quality (AMSTAR score 4), suggested that the risk of MS was largely dependent on influences occurring during the first two decades of life, and that risk of MS seemed to change between generations of migrants, although the authors of the review could not draw a firm conclusion due to obstacles in the interpretation of migrant studies, and the limited number of high-quality studies [51].

The papers already included in this previous review were not included in the present review, which identified four additional studies (Table 4). Three of them [52–54] reported results for age at migration and risk of MS of which two examined migration from high to low-risk area and one from low to a high-risk area. Only one study [55] addressed the question whether the risk of MS development changed in the second generation of migrants as opposed to the first generation. In the study among inhabitants of the French West Indies who returned to Martinique and Guadeloupe after migrating to France, a significantly increased standardized prevalence (SPR) and incidence ratio (SIR) for MS was found among West Indians who migrated before the age of 15 as compared to migration at older age (SPR 5.99, 95% CI 3.79–8.98 and SIR 4.05, 95% CI 2.17–6.83) [54]. In contrast, two later studies found no age dependence. The first of these studies found that immigrants from high-MS-risk areas, such as Ireland and the United Kingdom, who migrated to low-MS-risk area Australia, reduced their risk of developing MS independently of age at migration. Similarly, a case-control study that was performed in order to avoid bias related to the long duration of residence in Australia, did not find age related effects [52]. However, when McLeod et al. [53] later reanalyzed the same materials, they reported, in contrast to previous findings, that the risk of developing MS among immigrants from high to low-MS-risk zones was significantly lower among those who migrated at an age younger than 15 years (risk ratio 22.3/100,000 vs 45.3/100,000). Finally, a recent study from Norway compared the differences in MS prevalence between the first and second generation of migrants. The study found that the SPR of MS increased sharply in the second generation of migrants from Pakistan (SPR 1.62, 95% CI: 0.88–2.76) compared to the first generation migrants (SPR 0.13, 95% CI: 0.05–0.28), indicates an approximately twelve-fold increase in the risk of MS in the second generation of migrants [55].

The quality of the migrant studies could be assessed only for two of the four migration studies, both of which achieved a comparably good quality score but not higher because of their cross-sectional nature [54,55] (Table B in S2 Table). For the two other studies methodological quality could not be assessed as the methodology was insufficiently described [52,53].

## Discussion

Overall, the findings of this review suggest that an association between early life vitamin D availability and later risk of MS is possible, but also that there still is insufficient evidence to firmly conclude that low vitamin D availability in early life increases the risk of MS in adulthood as no intervention and few cohort studies, only, have been published. Furthermore, the results from the relatively few published observational studies were partly inconsistent, with some demonstrating significant inverse association between circulating 25(OH)D concentration in early life and risk of MS later in life, and some indicating no significant association.

Two of the included case-cohort studies had relatively large sample sizes and both examined dried blood spots collected from newborns for 25(OH)D for cases with MS onset and controls, but still found conflicting results. Both studies were from Scandinavia, and while the Danish study indicated a significant inverse association [26] the Swedish study found no association [24]. Conflict in findings may be due to the difference in sample storage temperature, as dried blood samples of the Swedish biobank were stored first at room temperature for six years and then at 4°C, while the Danish Newborn Screening biobank stored samples at -20°C temperature. A lower storage temperature may reduce the risk of degradation and maintain higher chemical stability of samples. The most comprehensive study to date in regards to control for confounding was that by Ueda and colleagues [24] who attempted to combine several additional risk factors in early (month of birth, latitude of birth, breastfeeding) and adult life (25-hydroxyvitamin D exposure, sun exposure, vitamin D intake from dairy products, fatty fish consumption, smoking, body mass index at 20 years of age) including ancestry, MS heredity and socioeconomic group. However, their results were essentially similar before and after control for the additional confounders. It is likely that interaction between genetic and environmental factors determine the risk of disease development, but exposure to Epstein-Barr virus, smoking, and latitude/vitamin D found to be most appropriate as these factors have shown the strongest evidence with MS development [56]. None of the studies adjusted for these factors simultaneously.

In contrast to the results from the population studies, the result from the many seasonal studies were relatively consistent, suggesting a lower occurrence of MS for late autumn born children on the Northern hemisphere. The overall excess of spring/summer MS births was further confirmed in the meta-analysis from the selected Northern hemisphere studies adding further support for a relation as meta-analyses allows greater precision in the effect size estimation. However, publication bias is always a possibility since non-significant results are less likely published [57], and publication bias may thus have resulted in an overestimation of the positive results found in the present review. We wanted to assess the potential for publication bias by inspection and testing asymmetry of funnel plots. However, although the visual inspection of the funnel plots suggested that there was no apparent tendency for publication bias related to the studies in our meta-analyses, less than 10 studies were included, and the power of the test was too low for distinguishing asymmetry. Therefore, it cannot be eliminated that the results of the meta-analysis were subject to publication bias, and the interpretation therefore needs to be done with caution. The present results from the seasonal studies are in agreement with previous systematic review findings by Torkildsen et al. [18] and Dobson et al. [30], both of which concluding that those born in autumn or winter, and thus exposed during early gestation to increased exposure to UV radiation, had a lower risk of later developing MS. Fewer studies were conducted in the Southern hemisphere and did not consistently identify a particular month or season of birth being of relevance for later MS development. Previous studies have been criticized for potentially being false positive due to inadequate control for confounders such as latitude and year of birth [58,59], adding caution to the interpretation of the positive findings. Still, very few (5/15) of the included studies adjusted for confounding from factors such as latitude and year of birth, and two studies, even indicated a significant month of birth effect after adjustment [45,47]. It is therefore still not clear if accounting for these sources of bias removes month of birth effect on MS development. Furthermore, the use of the average latitude as a proxy for the exposure of the population to UV radiation, and therefore for vitamin D formation, may be too imprecise. Many countries cover a wide range of latitudes amongst which the UV radiation may vary. Moreover, altitude above sea level of the place of residence of the subjects included in the studies, which is also a decisive factor of UV exposure, is not taken into consideration. Factors such as differences in temperature, sunscreen use along with clothing, which also may happen for e.g. religious reasons, might influence the seasonality effect on MS development. Also, potential explanations for seasonal risk variations could be seasonal differences to vitamin D exposure and differential responses of the body to sunlight exposure, which could affect the immune system during a vulnerable time of development. Other biological mechanisms for a month of birth effect have been described. For instance, a study examined blood samples from umbilical cords and found that those born in May had both lower levels of vitamin D and higher levels of autoreactive T-cells as opposed to those born in November [60]. Thus, high levels of autoreactive T-cells that attack the immune system and may trigger autoimmune disease, could also serve as a biological explanation for the month of birth relations with later MS. However, this needs further investigation. It is also possible that UVB is independently associated with risk of MS [61]. In addition, as MS is primarily considered to be an autoimmune disease, any season-dependent variation in regional patterns of contagious diseases may interfere with the development of autoreactive leukocytes triggering MS. Whether, in this regard, maternal infections during pregnancy may increase the risk of MS onset in the offspring remains to be clarified in future studies. One previous study examined this and did not see a significant association between infections during pregnancy and risk of MS development, possibly due to lack of power [38]. However, if such relation exists, the higher general risk of infection during winter (in January-March in the Western world where most studies were derived) may also provide reason why month of birth could influence MS incidence in the offspring. Another factor triggering the activity of autoreactive leukocytes responsible for MS onset are low levels of melatonin, which are lowest during spring [62]. Increased plasma levels of melatonin correlate with MS relapses [63]. Whether or not low levels of maternal melatonin during spring contributed to the elevated risk of MS onset in the offspring remains to be clarified in future studies. Finally, use of different statistical modeling may have led to differing results. Non-parametric tests such as Hewitt, Edwards’ and Cosinor tests may be more suitable in assessing seasonal patterns, whereas the Chi-square test may be less sensitive especially in case of a small sample size [36,64]. However, it is important to highlight that even though seasonal correlation analyses provide support for a link between MS onset and early life sun exposure, they do not provide evidence for an association between vitamin D and MS, as other seasonal factors such as infection, maternal nutrition, gene expression and other climatic factors also vary with season and may influence the risk of MS [64].

Finally, several of the migration studies also provided reasonably consistent results suggesting a difference in MS risk due to migration and between generations. However, even if, prevalence of a disease is a good epidemiological tool when measuring burden of disease, it is not necessarily the best tool for assessing the risk of disease due to migration. Also, as it was mentioned in a previous review [51], migrant studies may be subject to a quite high risk of selection bias, as migrants rarely are appropriate representatives of the population of the country of origin, as they usually differ by age, health and socioeconomic status.

While the comprehensive coverage of this systematic review is considered a strength, some limitations should also be mentioned. First, five of six population studies related to early life vitamin D and risk of MS in adulthood were from the Nordic countries and therefore the generalization to other populations of different ethnic backgrounds and locations might not be possible.

## Conclusion

Despite a growing interest in the relationship between vitamin D availability in early life and risk of MS in adulthood, the evidence from current studies remains suggestive. Nevertheless, this systematic review gives further support for an association between measured low 25(OH)D availability in early life and the later onset of MS. The findings from the season of birth, latitude and migration studies that are considered as proxy measures for vitamin D availability, provide some support for a possible association between low vitamin D in early life and later risk of MS, but the results from the population based studies are still relatively few and not entirely consistent, and intervention studies in the field are still absent. Overall, further research is therefore needed.

## Supporting information

S1 File PRISMA checklist. (DOC)

S2 File The review protocol which has been registered in PROSPERO International Prospective Register of Systematic Reviews. (PDF)

S3 File Newcastle—Ottawa quality assessment scale. (PDF)

S1 Fig Meta-analysis forest plots. (PDF)

S2 Fig The pooled estimates. Computed using the weights from the random effects meta-analysis.
(PDF)

S3 Fig Study specific estimates and the pooled estimates in one plot. (PDF)

S4 Fig The study specific SIRs and CI’s. (PDF)

S1 Table Search strategy documentation. (PDF)

S2 Table Methodological quality of included studies. (PDF)

S3 Table Confounders (Population studies). (PDF)

S4 Table Confounders (The season or the month of birth studies). (PDF)

## Abbreviations

25(OH)D: 25-hydroxyvitamin D
CNS: Central Nervous System
ICD: International Classification of Diseases
MS: Multiple Sclerosis

## References

[1] Harandi AA, Harandi AA, Pakdaman H, Sahraian MA. Vitamin D and multiple sclerosis. Iran J Neurol. Tehran University of Medical Sciences; 2014;13: 1–6. PMC3968350 24800040

[2] Harbo HF, Gold R, Tintoré M. Sex and gender issues in multiple sclerosis. Ther Adv Neurol Disord. SAGE Publications; 2013;6: 237–48. 10.1177/1756285613488434 23858327 PMC3707353

[3] World Health Organization. Neurological disorders: public health challenges. WHO Press; 2006.

[4] Sayetta RB. Theories of the etiology of multiple sclerosis: a critical review. J Clin Lab Immunol. 1986;21: 55–70. 3102746

[5] Alonso A, Hernan MA. Temporal trends in the incidence of multiple sclerosis: a systematic review. Neurology. 2008;71: 129–135. 10.1212/01.wnl.0000316802.35974.34 18606967 PMC4109189

[6] Kurtzke JF. A reassessment of the distribution of multiple sclerosis. Acta Neurol Scand. John Wiley & Sons, Ltd (10.1111); 1975;51: 110–136. 10.1111/j.1600-0404.1975.tb01364.x 46682

[7] Pakpoor J, Giovannoni G, Ramagopalan S V. Epstein–Barr virus and multiple sclerosis: association or causation? Expert Rev Neurother. 2013;13: 287–297. 10.1586/ern.13.6 23448218

[8] Wingerchuk DM. Smoking: effects on multiple sclerosis susceptibility and disease progression. Ther Adv Neurol Disord. SAGE Publications; 2012;5: 13–22. 10.1177/1756285611425694 22276073 PMC3251901

[9] Munger KL, Bentzen J, Laursen B, Stenager E, Koch-Henriksen N, Sørensen TI, et al Childhood body mass index and multiple sclerosis risk: a long-term cohort study. Mult Scler J. 2013;19: 1323–1329. 10.1177/1352458513483889 23549432 PMC4418015

[10] Correale J, Balbuena Aguirre ME, Farez M. Body Mass Index and Multiple Sclerosis Risk. The Role of Leptin (S24.004). Neurol. 2014;82.

[11] Alharbi F. Update in vitamin D and multiple sclerosis. Neurosciences. 2015;20: 329–335. 26492110 10.17712/nsj.2015.4.20150357 PMC4727614

[12] Pakpoor J, Ramagopalan S. Evidence for an Association Between Vitamin D and Multiple Sclerosis. 2014 pp. 105–115. 10.1007/7854_2014_358 25502544

[13] Chaudhuri A. Why we should offer routine vitamin D supplementation in pregnancy and childhood to prevent multiple sclerosis. Med Hypotheses. 2005;64: 608–618. 10.1016/j.mehy.2004.06.022 15617877

[14] Kaludjerovic J, Vieth R. Relationship between vitamin D during perinatal development and health. J Midwifery Womens Health. 2010;55: 550–560. 10.1016/j.jmwh.2010.02.016 20974417

[15] Eyles DW, Feron F, Cui X, Kesby JP, Harms LH, Ko P, et al Developmental vitamin D deficiency causes abnormal brain development. Psychoneuroendocrinology. 2009;34 Suppl 1: 247 10.1016/j.psyneuen.2009.04.015 19500914

[16] Krementsov DN, Teuscher C. Environmental factors acting during development to influence MS risk: insights from animal studies. Mult Scler J. 2013;19: 1684–1689. 10.1177/1352458513506954 24077054 PMC3833345

[17] Moher D, Shamseer L, Clarke M, Ghersi D, Liberati A, Petticrew M, et al Preferred reporting items for systematic review and meta-analysis protocols (PRISMA-P) 2015 statement. Syst Rev. 2015;4: 1 10.1186/2046-4053-4-1 25554246 PMC4320440

[18] Torkildsen O, Grytten N, Aarseth J, Myhr KM, Kampman MT. Month of birth as a risk factor for multiple sclerosis: an update. Acta Neurol Scand. John Wiley & Sons A/S; 2012;(195):58–6: 58–62. 10.1111/ane.12040 23278658

[19] Sharif MO, Janjua-Sharif FN, Sharif FNJ, Ali H, Ahmed F. Systematic reviews explained: AMSTAR-how to tell the good from the bad and the ugly. Oral Health Dent Manag. 2013;12: 9–16. 23474576

[20] Higgins JPT, Thompson SG. Quantifying heterogeneity in a meta-analysis. Stat Med. 2002;21: 1539–1558. 10.1002/sim.1186 12111919

[21] R Core Team. R: A language and environment for statistical computing. R Foundation for Statistical Computing, Vienna, Austria [Internet]. 2018 https://www.r-project.org/.

[22] Schwarzer G. meta : An R Package for Meta-Analysis. 2007;

[23] Salzer J, Hallmans G, Nystrom M, Stenlund H, Wadell G, Sundstrom P. Vitamin D as a protective factor in multiple sclerosis. Neurology. 2012;79: 2140–2145. 10.1212/WNL.0b013e3182752ea8 23170011

[24] Ueda P, Rafatnia F, Baarnhielm M, Frobom R, Korzunowicz G, Lonnerbro R, et al Neonatal vitamin D status and risk of multiple sclerosis. Ann Neurol. American Neurological Association; 2014;76: 338–346. 10.1002/ana.24210 24985080

[25] Munger KL, Aivo J, Hongell K, Soilu-Hanninen M, Surcel HM, Ascherio A. Vitamin D Status During Pregnancy and Risk of Multiple Sclerosis in Offspring of Women in the Finnish Maternity Cohort. JAMA Neurol. 2016;73: 515–519. 10.1001/jamaneurol.2015.4800 26953778 PMC4861670

[26] Nielsen NM, Munger KL, Koch-Henriksen N, Hougaard DM, Magyari M, Jorgensen KT, et al Neonatal vitamin D status and risk of multiple sclerosis: A population-based case-control study. Neurology. 2016; 10.1212/WNL.0000000000003454 27903815 PMC5200855

[27] Cortese M, Riise T, Bjørnevik K, Holmøy T, Kampman MT, Magalhaes S, et al Timing of use of cod liver oil, a vitamin D source, and multiple sclerosis risk: The EnvIMS study. Mult Scler. SAGE Publications; 2015;21: 1856–64. 10.1177/1352458515578770 25948625 PMC4657387

[28] Mirzaei F, Michels KB, Munger K, O’Reilly E, Chitnis T, Forman MR, et al Gestational vitamin D and the risk of multiple sclerosis in offspring. Ann Neurol. American Neurological Association; 2011;70: 30–40. 10.1002/ana.22456 21786297 PMC3205990

[29] Watson PE, McDonald BW. Seasonal variation of nutrient intake in pregnancy: effects on infant measures and possible influence on diseases related to season of birth. Eur J Clin Nutr. 2007;61: 1271–1280. 10.1038/sj.ejcn.1602644 17299488

[30] Dobson R, Giovannoni G, Ramagopalan S. The month of birth effect in multiple sclerosis: systematic review, meta-analysis and effect of latitude. J Neurol Neurosurg Psychiatry. 2013;84: 427–432. 10.1136/jnnp-2012-303934 23152637

[31] Becker J, Callegaro D, Lana-Peixoto MA, Ferreira ML, Melo A, Diniz da Gama P, et al Season of birth as a risk factor for multiple sclerosis in Brazil. J Neurol Sci. Elsevier B.V; 2013;329: 6–10. 10.1016/j.jns.2013.03.001 23597669

[32] Barros P, de Sá JM, Sá MJ. Month of birth and risk of multiple sclerosis in a Portuguese population. Clin Neurol Neurosurg. 2013;115: 1762–5. 10.1016/j.clineuro.2013.04.007 23643141

[33] Torkildsen O, Aarseth J, Benjaminsen E, Celius E, Holmoy T, Kampman MT, et al Month of birth and risk of multiple sclerosis: confounding and adjustments. Ann Clin Transl Neurol. 2014;1: 141–144. 10.1002/acn3.37 25356394 PMC4212485

[34] Akhtar S, Alroughani R, Al-Shammari A, Al-Abkal J, Ayad Y. Non-parametric analysis of seasonality in birth and multiple sclerosis risk in second generation of migrants in Kuwait. BMC Neurol. BioMed Central; 2014;14: 170 10.1186/s12883-014-0170-7 25154872 PMC4236647

[35] Akhtar S, Alroughani R, Al-Shammari A, Al-Abkal J, Ayad Y. Month of birth and risk of multiple sclerosis in Kuwait: a population-based registry study. Mult Scler. 2015;21: 147–154. 10.1177/1352458514541578 25092768

[36] Sidhom Y, Kacem I, Bayoudh L, Ben Djebara M, Hizem Y, Ben Abdelfettah S, et al Season of birth and multiple sclerosis in Tunisia. Mult Scler Relat Disord. Elsevier B.V; 2015;4: 491–494. 10.1016/j.msard.2015.08.002 26590652

[37] Villar-Quiles RN, Matias-Guiu JA, Ortega G, Gonzalez-Suarez I, Oreja-Guevara C, Matias-Guiu J. Analysis of the Relationship between the Month of Birth and Risk of Multiple Sclerosis in a Spanish Population. Eur Neurol. 2016;76: 202–209. 10.1159/000449246 27721317

[38] Gardener H, Munger KL, Chitnis T, Michels KB, Spiegelman D, Ascherio A. Prenatal and perinatal factors and risk of multiple sclerosis. Epidemiology. 2009;20: 611–618. 10.1097/EDE.0b013e31819ed4b9 19333127 PMC3132937

[39] Fragoso YD, Adoni T, Almeida SM, Alves-Leon S V, Arruda WO, Barbagelata-Aguero F, et al Multiple sclerosis in South America: month of birth in different latitudes does not seem to interfere with the prevalence or progression of the disease. Arq Neuropsiquiatr. 2013;71: 573–579. 10.1590/0004-282X20130098 24141434

[40] Tolou-Ghamari Z, Shygannejad V, Ashtari F, Chitsaz A, Palizban AA. Preliminary analysis of month of birth in Iranian/Isfahan patients with multiple sclerosis. Adv Biomed Res. 2015;4: 9175162543. eCollection 2015. 10.4103/2277-9175.162543 26436080 PMC4581128

[41] Poorolajal J, Mazdeh M, Saatchi M, Talebi Ghane E, Biderafsh A, Lotfi B, et al Multiple Sclerosis Associated Risk Factors: A Case-Control Study. Iran J Public Health. 2015;44: 1498–505. 26744707 PMC4703229

[42] Vieth Streym S, Rejnmark L, Mosekilde L, Vestergaard P. No effect of season of birth on risk of type 1 diabetes, cancer, schizophrenia and ischemic heart disease, while some variations may be seen for pneumonia and multiple sclerosis. Dermatoendocrinol. 2013;5: 309–316. 10.4161/derm.22779 24194971 PMC3772919

[43] Balbuena LD, Middleton RM, Tuite-Dalton K, Pouliou T, Williams KE, Noble GJ. Sunshine, Sea, and Season of Birth: MS Incidence in Wales. Burne THJ, editor. PLoS One. Public Library of Science; 2016;11: e0155181 10.1371/journal.pone.0155181 27182982 PMC4868284

[44] Verheul F, Smolders J, Trojano M, Lepore V, Zwanikken C, Amato MP, et al Fluctuations of MS births and UV-light exposure. Acta Neurol Scand. John Wiley & Sons A/S; 2013;127: 301–308. 10.1111/ane.12007 22970985

[45] Rodríguez Cruz PM, Matthews L, Boggild M, Cavey A, Constantinescu CS, Evangelou N, et al Time- and Region-Specific Season of Birth Effects in Multiple Sclerosis in the United Kingdom. JAMA Neurol. 2016;73: 954 10.1001/jamaneurol.2016.1463 27366989

[46] Tolou-Ghamari Z, Shygannejad V, Ashtari F, Chitsaz A, Palizban AA. Preliminary analysis of month of birth in Iranian/Isfahan patients with multiple sclerosis. Adv Biomed Res. 2015;4: 9175162543. eCollection 2015. 10.4103/2277-9175.162543 26436080 PMC4581128

[47] Torkildsen O, Aarseth J, Benjaminsen E, Celius E, Holmøy T, Kampman MT, et al Month of birth and risk of multiple sclerosis: confounding and adjustments. Ann Clin Transl Neurol. Wiley-Blackwell; 2014;1: 141–4. 10.1002/acn3.37 25356394 PMC4212485

[48] Akhtar S, Alroughani R, Al-Shammari A, Al-Abkal J, Ayad Y. Month of birth and risk of multiple sclerosis in Kuwait: A population-based registry study. Mult Scler J. 2015;21: 147–154. 10.1177/1352458514541578 25092768

[49] Sidhom Y, Kacem I, Bayoudh L, Ben Djebara M, Hizem Y, Ben Abdelfettah S, et al Season of birth and multiple sclerosis in Tunisia. Mult Scler Relat Disord. Elsevier B.V; 2015;4: 491–494. 10.1016/j.msard.2015.08.002 26590652

[50] Villar-Quiles RN, Matías-Guiu JA, Ortega G, González-Suárez I, Oreja-Guevara C, Matías-Guiu J. Analysis of the Relationship between the Month of Birth and Risk of Multiple Sclerosis in a Spanish Population. Eur Neurol. 2016;76: 202–209. 10.1159/000449246 27721317

[51] Gale CR, Martyn CN. Migrant studies in multiple sclerosis. Prog Neurobiol. 47: 425–48. 8966212

[52] Hammond SR, English DR, McLeod JG. The age-range of risk of developing multiple sclerosis: evidence from a migrant population in Australia. Brain. 2000;123 (Pt 5: 968–974. 10775541 10.1093/brain/123.5.968

[53] McLeod JG, Hammond SR, Kurtzke JF. Migration and multiple sclerosis in immigrants to Australia from United Kingdom and Ireland: a reassessment. I. Risk of MS by age at immigration. J Neurol. 2011;258: 1140–1149. 10.1007/s00415-010-5898-4 21264474 PMC3101360

[54] Cabre P, Signate A, Olindo S, Merle H, Caparros-Lefebvre D, Bera O, et al Role of return migration in the emergence of multiple sclerosis in the French West Indies. Brain. 2005;128: 2899–2910. awh624 10.1093/brain/awh624 16183661

[55] Berg-Hansen P, Moen SM, Sandvik L, Harbo HF, Bakken IJ, Stoltenberg C, et al Prevalence of multiple sclerosis among immigrants in Norway. Mult Scler. 2015;21: 695–702. 10.1177/1352458514554055 25344371

[56] Ramagopalan SV, Dobson R, Meier UC, Giovannoni G. Multiple sclerosis: risk factors, prodromes, and potential causal pathways. Lancet Neurol. England; 2010;9: 727–739. 10.1016/S1474-4422(10)70094-6 20610348

[57] Duyx B, Urlings MJE, Swaen GMH, Bouter LM, Zeegers MP. Scientific citations favor positive results: a systematic review and meta-analysis. J Clin Epidemiol. United States; 2017;88: 92–101. 10.1016/j.jclinepi.2017.06.002 28603008

[58] Fiddes B, Wason J, Kemppinen A, Ban M, Compston A, Sawcer S. Confounding underlies the apparent month of birth effect in multiple sclerosis. Ann Neurol. Wiley-Blackwell; 2013;73: 714–20. 10.1002/ana.23925 23744589 PMC3748787

[59] Fiddes B, Wason J, Sawcer S. Confounding in association studies: month of birth and multiple sclerosis. J Neurol. Springer; 2014;261: 1851–1856. 10.1007/s00415-014-7241-y 24413643 PMC4192561

[60] Disanto G, Watson CT, Meier UC, Ebers GC, Giovannoni G, Ramagopalan SV. Month of birth and thymic output. JAMA neurology. 2013 pp. 527–528. 10.1001/jamaneurol.2013.2116 23568650

[61] Åivo J. Vitamin D in the prevention and treatment of multiple sclerosis. University of Turku; 2017.

[62] Srinivasan V, Spence DW, Trakht I, Pandi-Perumal SR, Cardinali DP, Maestroni GJ. Immunomodulation by melatonin: its significance for seasonally occurring diseases. Neuroimmunomodulation. Switzerland; 2008;15: 93–101. 10.1159/000148191 18679047

[63] Farez MF, Mascanfroni ID, Mendez-Huergo SP, Yeste A, Murugaiyan G, Garo LP, et al Melatonin Contributes to the Seasonality of Multiple Sclerosis Relapses. Cell. United States; 2015;162: 1338–1352. 10.1016/j.cell.2015.08.025 26359987 PMC4570563

[64] Disanto G, Chaplin G, Morahan JM, Giovannoni G, Hypponen E, Ebers GC, et al Month of birth, vitamin D and risk of immune-mediated disease: a case control study. BMC Med. 2012;10: 69 10.1186/1741-7015-10-69 22764877 PMC3395583

