# Supplementary material for: Vitamin D in early life and later risk of multiple sclerosis—A systematic review, meta-analysis
Source: PLoS One. 2019 Aug 27;14(8):e0221645. doi: 10.1371/journal.pone.0221645 (PMC6711523; doi:10.1371/journal.pone.0221645)
Supplement: S2 File — (PDF) [file pone.0221645.s002.pdf]

Systematic review

1. * Review title.

Give the working title of the review, for example the one used for obtaining funding. Ideally the title should state succinctly the interventions or exposures being reviewed and the associated health or social problems. Where appropriate, the title should use the PI(E)COS structure to contain information on the Participants, Intervention (or Exposure) and Comparison groups, the Outcomes to be measured and Study designs to be included.

Vitamin D in early life and later risk of multiple sclerosis: a systematic review

2. Original language title.

For reviews in languages other than English, this field should be used to enter the title in the language of the review. This will be displayed together with the English language title.

3. * Anticipated or actual start date.

Give the date when the systematic review commenced, or is expected to commence.

23/07/2016

4. * Anticipated completion date.

Give the date by which the review is expected to be completed.

23/01/2017

5. * Stage of review at time of this submission.

Indicate the stage of progress of the review by ticking the relevant Started and Completed boxes. Additional information may be added in the free text box provided.
Please note: Reviews that have progressed beyond the point of completing data extraction at the time of initial registration are not eligible for inclusion in PROSPERO. Should evidence of incorrect status and/or completion date being supplied at the time of submission come to light, the content of the PROSPERO record will be removed leaving only the title and named contact details and a statement that inaccuracies in the stage of the review date had been identified.
This field should be updated when any amendments are made to a published record and on completion and publication of the review. If this field was pre-populated from the initial screening questions then you are not able to edit it until the record is published.

The review has not yet started: No

Review stage	Started	Completed
Preliminary searches	Yes	Yes
Piloting of the study selection process	Yes	Yes
Formal screening of search results against eligibility criteria	Yes	Yes
Data extraction	Yes	Yes
Risk of bias (quality) assessment	Yes	Yes
Data analysis	Yes	Yes

Provide any other relevant information about the stage of the review here (e.g. Funded proposal, protocol not yet finalised).

6. * Named contact.

The named contact acts as the guarantor for the accuracy of the information presented in the register record.

Professor Heitmann

Email salutation (e.g. "Dr Smith" or "Joanne") for correspondence:

7. * Named contact email.

Give the electronic mail address of the named contact.

berit.lilienthal.heitmann@regionh.dk

8. Named contact address

Give the full postal address for the named contact.

Nordre Fasanvej 57, Hovedvejen, entrance 5, ground floor

2000 Frederiksberg - Denmark

9. Named contact phone number.

Give the telephone number for the named contact, including international dialling code.

+4538163070

10. * Organisational affiliation of the review.

Full title of the organisational affiliations for this review and website address if available. This field may be completed as 'None' if the review is not affiliated to any organisation.

Research Unit for Dietary Studies at The Parker Institute

Organisation web address:

http://kostforskning.dk/om-eek/

11. * Review team members and their organisational affiliations.

Give the title, first name, last name and the organisational affiliations of each member of the review team. Affiliation refers to groups or organisations to which review team members belong.

Professor Berit Heitmann.
Mrs Kamila Ismailova.
Mrs Pratiksha Poudel.

12. * Funding sources/sponsors.

Give details of the individuals, organizations, groups or other legal entities who take responsibility for initiating, managing, sponsoring and/or financing the review. Include any unique identification numbers assigned to the review by the individuals or bodies listed.

The Programme Commission on Health, Food, and Welfare under the Danish Council for Strategic research

(grant number 0603-00453B)

13. * Conflicts of interest.

List any conditions that could lead to actual or perceived undue influence on judgements concerning the main topic investigated in the review.

None

14. Collaborators.

Give the name and affiliation of any individuals or organisations who are working on the review but who are not listed as review team members.

15. * Review question.

State the question(s) to be addressed by the review, clearly and precisely. Review questions may be specific or broad. It may be appropriate to break very broad questions down into a series of related more specific questions. Questions may be framed or refined using PI(E)COS where relevant.

Can an increased vitamin D availability in early life contribute to a reduced risk of MS in adulthood?

Is the season or the month of birth related to subsequent risk of MS?

Does the season or month of birth patterns in MS differ according to different latitudinal gradients?

Does early or late age at migration relate to MS?

Does the risk of MS differ in the second generation of migrants as opposed to the first generation?

16. * Searches.

Give details of the sources to be searched, search dates (from and to), and any restrictions (e.g. language or publication period). The full search strategy is not required, but may be supplied as a link or attachment.

The following databases will be searched: PubMed/MEDLINE for relevant studies and Web of Science for

the citation search, without restrictions on publication period.

Articles published in English will be considered.

We will also hand search the references of included studies to supplement the database search. The

searches will be re-run before the final analysis and further studies retrieved for inclusion.

17. URL to search strategy.

Give a link to a published pdf/word document detailing either the search strategy or an example of a search

strategy for a specific database if available (including the keywords that will be used in the search strategies), or upload your search strategy.Do NOT provide links to your search results.

http://www.crd.york.ac.uk/PROSPEROFILES/43229_STRATEGY_20160620.pdf

Alternatively, upload your search strategy to CRD in pdf format. Please note that by doing so you are consenting to the file being made publicly accessible.

Yes I give permission for this file to be made publicly available

18. * Condition or domain being studied.

Give a short description of the disease, condition or healthcare domain being studied. This could include health and wellbeing outcomes.

In this review, we will study multiple sclerosis (MS) that is a chronic demyelinating disease affecting the Central Nervous System (CNS). In recent years, vitamin d insufficiency has been suggested as one of the predisposing factors for development of MS. Research suggests that low levels of vitamin d may be of particular concern during fetal life and may increase susceptibility to develop MS later in life. Furthermore, migrations, season of birth and latitudinal gradient may be other possible correlates of multiple sclerosis.

19. * Participants/population.

Give summary criteria for the participants or populations being studied by the review. The preferred format includes details of both inclusion and exclusion criteria.

Inclusion criteria:

For vitamin D analysis: pregnant women without MS and neonates (from birth to 1 month);

For season/month of birth and migration analysis: adults of any age group with MS;

The region in which the studies have been conducted is important to note in order to understand the possible relationships between the season/month of the birth effect and MS susceptibility in different latitudinal gradients. Therefore, there will be no limitations on study settings.

Exclusion criteria:

Pregnant women with MS and animals.

20. * Intervention(s), exposure(s).

Give full and clear descriptions or definitions of the nature of the interventions or the exposures to be reviewed.

Exposure 1: vitamin D;

Exposure 2: season/month of birth effect;

Exposure 3: migration (age at migration and risk between generations of migrants).

21. * Comparator(s)/control.

Where relevant, give details of the alternatives against which the main subject/topic of the review will be compared (e.g. another intervention or a non-exposed control group). The preferred format includes details of both inclusion and exclusion criteria.

We will compare with control group and also include studies that have no comparator.

22. * Types of study to be included.

Give details of the types of study (study designs) eligible for inclusion in the review. If there are no restrictions on the types of study design eligible for inclusion, or certain study types are excluded, this should be stated. The preferred format includes details of both inclusion and exclusion criteria.

Inclusion criteriaWe will include intervention studies, human observational studies (cross sectional and prospective/retrospective), historical comparative cohorts and if appropriate, recent systematic reviews.Exclusion criteriaWe will exclude case series, letters, commentaries, editorials, reports and animal studies.

23. Context.

Give summary details of the setting and other relevant characteristics which help define the inclusion or exclusion criteria.

This review will not be restricted to any specific context.

24. * Main outcome(s).

Give the pre-specified main (most important) outcomes of the review, including details of how the outcome is defined and measured and when these measurement are made, if these are part of the review inclusion criteria.

Multiple sclerosis

Timing and effect measures

Early life influences

25. * Additional outcome(s).

List the pre-specified additional outcomes of the review, with a similar level of detail to that required for main outcomes. Where there are no additional outcomes please state 'None' or 'Not applicable' as appropriate to the review

None

Timing and effect measures

26. * Data extraction (selection and coding).

Give the procedure for selecting studies for the review and extracting data, including the number of researchers involved and how discrepancies will be resolved. List the data to be extracted.

Studies will be screened for titles and abstracts independently by two review authors in order to identify studies that meet the inclusion criteria. Afterwards, full texts of potentially eligible studies will be retrieved for full text review and assessed for eligibility by two review authors. Disagreements will be resolved through discussion with a third reviewer.

A standardized form will be used for data extraction from included studies to assess study quality. Extracted information from studies will include: study objectives, study characteristics (study design, country, year of publication and sample size), participant characteristics (age and gender) exposure/intervention, methodological quality and main findings of results. At the level of the systematic review, we will extract data

on author name, year of publication, included studies, methodological quality and main findings.

27. * Risk of bias (quality) assessment.

State whether and how risk of bias will be assessed (including the number of researchers involved and how discrepancies will be resolved), how the quality of individual studies will be assessed, and whether and how this will influence the planned synthesis.

Two review authors will independently assess the risk of bias in included studies. Cohort and case-control studies will be assessed for quality using the Newcastle-Ottawa Scale, while systematic reviews will be assessed using the AMSTAR Score.

28. * Strategy for data synthesis.

Give the planned general approach to synthesis, e.g. whether aggregate or individual participant data will be used and whether a quantitative or narrative (descriptive) synthesis is planned. It is acceptable to state that a quantitative synthesis will be used if the included studies are sufficiently homogenous.

Results of the systematic review will be presented in accordance with PRISMA recommendations. A flow diagram will provide a summary of each step during the search procedures. Summary and data from included studies will be presented through tables and described in the results section. Heterogeneity of methods and population will also be assessed and described. If the heterogeneity of studies varies highly, meta-analysis will not be performed as the feasibility is likely to be limited.

29. * Analysis of subgroups or subsets.

Give details of any plans for the separate presentation, exploration or analysis of different types of participants (e.g. by age, disease status, ethnicity, socioeconomic status, presence or absence or co-morbidities); different types of intervention (e.g. drug dose, presence or absence of particular components of intervention); different settings (e.g. country, acute or primary care sector, professional or family care); or different types of study (e.g. randomised or non-randomised).

Not planned

30. * Type and method of review.

Select the type of review and the review method from the lists below. Select the health area(s) of interest for your review.

Type of review

Cost effectiveness
No

Diagnostic
No

Epidemiologic
Yes

Individual patient data (IPD) meta-analysis
No

Intervention
No

Meta-analysis
No

Methodology

No

Narrative synthesis
No

Network meta-analysis
No

Pre-clinical
No

Prevention
No

Prognostic
No

Prospective meta-analysis (PMA)
No

Review of reviews
No

Service delivery
No

Synthesis of qualitative studies
No

Systematic review
Yes

Other
No

Health area of the review

Alcohol/substance misuse/abuse
No

Blood and immune system
No

Cancer
No

Cardiovascular
No

Care of the elderly
No

Child health
No

Complementary therapies
No

Crime and justice
No

Dental
No

Digestive system
No

Ear, nose and throat
No

Education
No

Endocrine and metabolic disorders

No

Eye disorders
No

General interest
No

Genetics
No

Health inequalities/health equity
No

Infections and infestations
No

International development
No

Mental health and behavioural conditions
No

Musculoskeletal
No

Neurological
No

Nursing
No

Obstetrics and gynaecology
No

Oral health
No

Palliative care
No

Perioperative care
No

Physiotherapy
No

Pregnancy and childbirth
No

Public health (including social determinants of health)
No

Rehabilitation
No

Respiratory disorders
No

Service delivery
No

Skin disorders
No

Social care
No

Surgery
No

Tropical Medicine
No

Urological
No

Wounds, injuries and accidents
No

Violence and abuse
No

31. Language.

Select each language individually to add it to the list below, use the bin icon to remove any added in error.
English

There is an English language summary.

32. Country.

Select the country in which the review is being carried out from the drop down list. For multi-national collaborations select all the countries involved.

Denmark

33. Other registration details.

Give the name of any organisation where the systematic review title or protocol is registered (such as with The Campbell Collaboration, or The Joanna Briggs Institute) together with any unique identification number assigned. (N.B. Registration details for Cochrane protocols will be automatically entered). If extracted data will be stored and made available through a repository such as the Systematic Review Data Repository (SRDR), details and a link should be included here. If none, leave blank.

34. Reference and/or URL for published protocol.

Give the citation and link for the published protocol, if there is one

Give the link to the published protocol.

Alternatively, upload your published protocol to CRD in pdf format. Please note that by doing so you are consenting to the file being made publicly accessible.

Yes I give permission for this file to be made publicly available

Please note that the information required in the PROSPERO registration form must be completed in full even if access to a protocol is given.

35. Dissemination plans.

Give brief details of plans for communicating essential messages from the review to the appropriate audiences.

Do you intend to publish the review on completion?

Yes

36. Keywords.

Give words or phrases that best describe the review. Separate keywords with a semicolon or new line. Keywords will help users find the review in the Register (the words do not appear in the public record but are included in searches). Be as specific and precise as possible. Avoid acronyms and abbreviations unless these are in wide use.

Vitamin D and multiple sclerosis

season or month of birth

migration

early life vitamin d

MS

37. Details of any existing review of the same topic by the same authors.

Give details of earlier versions of the systematic review if an update of an existing review is being registered, including full bibliographic reference if possible.

38. * Current review status.

Review status should be updated when the review is completed and when it is published. For newregistrations the review must be Ongoing.
Please provide anticipated publication date

Review_Ongoing

39. Any additional information.

Provide any other information the review team feel is relevant to the registration of the review.

40. Details of final report/publication(s).

This field should be left empty until details of the completed review are available.

Give the link to the published review.
