# Supplementary material for: Vitamin D in early life and later risk of multiple sclerosis—A systematic review, meta-analysis
Source: PLoS One. 2019 Aug 27;14(8):e0221645. doi: 10.1371/journal.pone.0221645 (PMC6711523; doi:10.1371/journal.pone.0221645)
Supplement: S3 File — (PDF) [file pone.0221645.s003.pdf]

S1 File. Newcastle - Ottawa quality assessment scale

CASE CONTROL STUDIES

Selection

1) Is the case definition adequate?

a) yes, with independent validation ✵

b) yes, eg record linkage or based on self reports

c) no description

2) Representativeness of the cases

a) consecutive or obviously representative series of cases ✵

b) potential for selection biases or not stated

3) Selection of Controls

a) community controls ✵

b) hospital controls

c) no description

4) Definition of Controls

a) no history of disease (endpoint) ✵

b) no description of source

Comparability

1) Comparability of cases and controls on the basis of the design or analysis

a) study controls for ________________ ✵

b) study controls for any additional factor ✵

Exposure

1) Ascertainment of exposure

a) secure record (eg surgical records) ✵

b) structured interview where blind to case/control status ✵

c) interview not blinded to case/control status

d) written self report or medical record only

e) no description

2) Same method of ascertainment for cases and controls

a) yes ✵

b) no

3) Non-Response rate

a) same rate for both groups ✵

b) non respondents described

c) rate different and no designation

COHORT STUDIES

Selection

1) Representativeness of the exposed cohort

a) truly representative of the average _______________ in the community ✵

b) somewhat representative of the average ______________ in the community ✵

c) selected group of users eg nurses, volunteers

d) no description of the derivation of the cohort

2) Selection of the non exposed cohort

a) drawn from the same community as the exposed cohort ✵

b) drawn from a different source

c) no description of the derivation of the non exposed cohort

3) Ascertainment of exposure

a) secure record (eg surgical records) ✵

b) structured interview ✵

c) written self report

d) no description

4) Demonstration that outcome of interest was not present at start of study

a) yes ✵

b) no

Comparability

1) Comparability of cohorts on the basis of the design or analysis

a) study controls for _____________ ✵

b) study controls for any additional factor ✵

Outcome

1) <u>Assessment of outcome</u>

a) independent blind assessment ✵

b) record linkage ✵

c) self report

d) no description

2) Was follow-up long enough for outcomes to occur

a) yes ✵

b) no

3) Adequacy of follow up of cohorts

a) complete follow up - all subjects accounted for ✵

b) subjects lost to follow up unlikely to introduce bias - small number lost - > __20__ %, or description provided of those lost ✵

c) no description of those lost

d) no statement

ADAPTED FOR CROSS-SECTIONAL STUDIES

Selection: (Maximum 3 stars)

1) Representativeness of the sample:

a) Truly representative of the average in the target population. ✵ (all subjects or random sampling)

b) Somewhat representative of the average in the target population. ✵ (non-random sampling)

c) Selected group of users.

d) No description of the sampling strategy.

2) Non-respondents:

a) Comparability between respondents and non-respondents characteristics is established, and the response rate is satisfactory. ✵
b) The response rate is unsatisfactory, or the comparability between respondents and non-respondents is unsatisfactory.
c) No description of the response rate or the characteristics of the responders and the non-responders.

3) Ascertainment of the exposure (risk factor):

a) Validated measurement tool. ✵

b) Non-validated measurement tool, but the tool is available or described.

c) No description of the measurement tool.

Comparability: (Maximum 2 stars)

1) The subjects in different outcome groups are comparable, based on the study design or analysis. Confounding factors are controlled.

a) The study controls for the most important factor.✵

b) The study control for any additional factor. ✵

Outcome: (Maximum 2 stars)

1) Assessment of the outcome:

a) Independent blind assessment. ✵

b) Record linkage. ✵

c) Self report.

d) No description.

2) Statistical test:

a) The statistical test used to analyze the data is clearly described and appropriate, and the

measurement of the association is presented, including confidence intervals and the probability level (p value). ✵

b) The statistical test is not appropriate, not described or incomplete.

The scale assessing cross-sectional studies was a modified version of the Newcastle-Ottawa Scale, it was also used by several other studies that found it appropriate for assessing the quality of cross-sectional studies.
