# Supplementary figures and images for: Vitamin D in early life and later risk of multiple sclerosis—A systematic review, meta-analysis
Source: PLoS One. 2019 Aug 27;14(8):e0221645. doi: 10.1371/journal.pone.0221645 (PMC6711523; doi:10.1371/journal.pone.0221645)

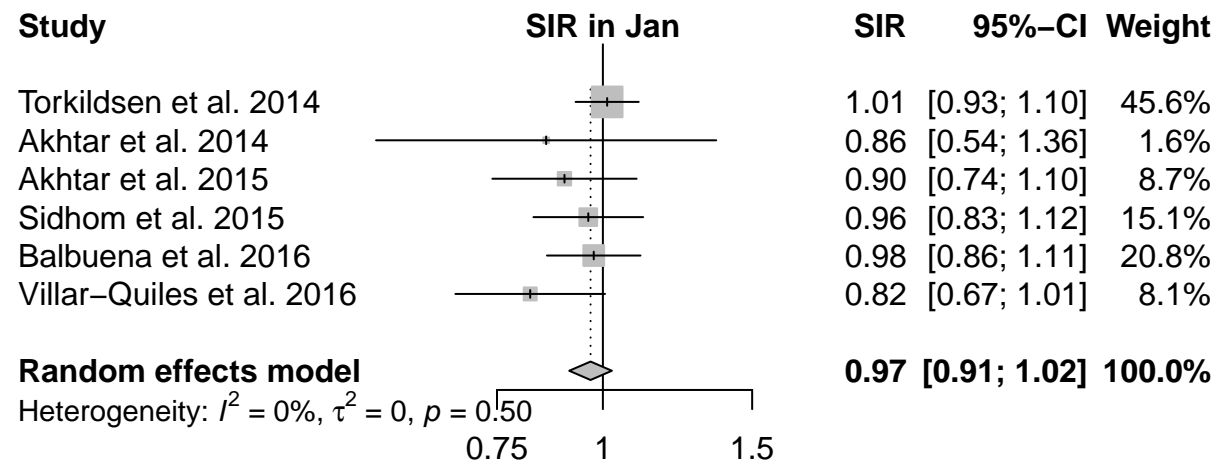

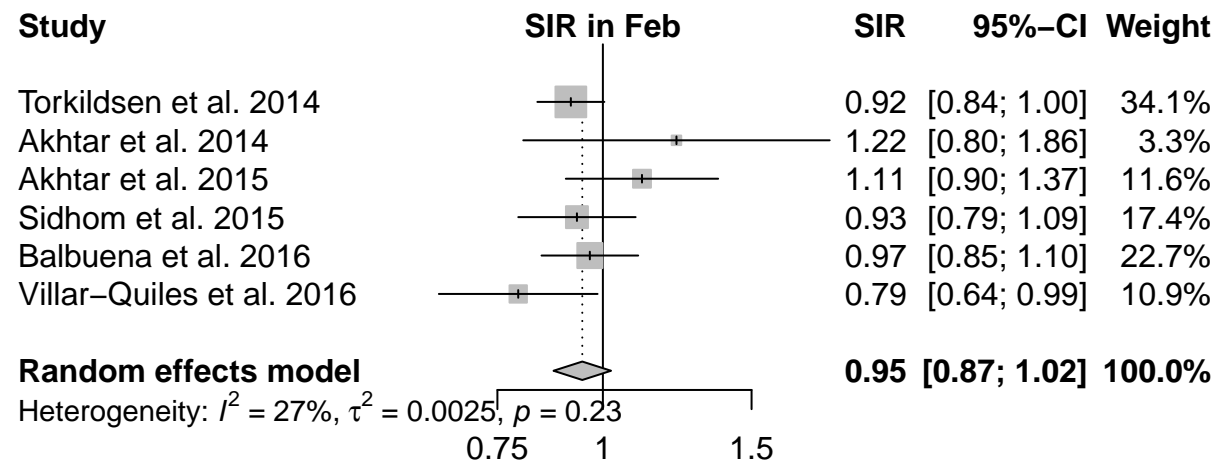

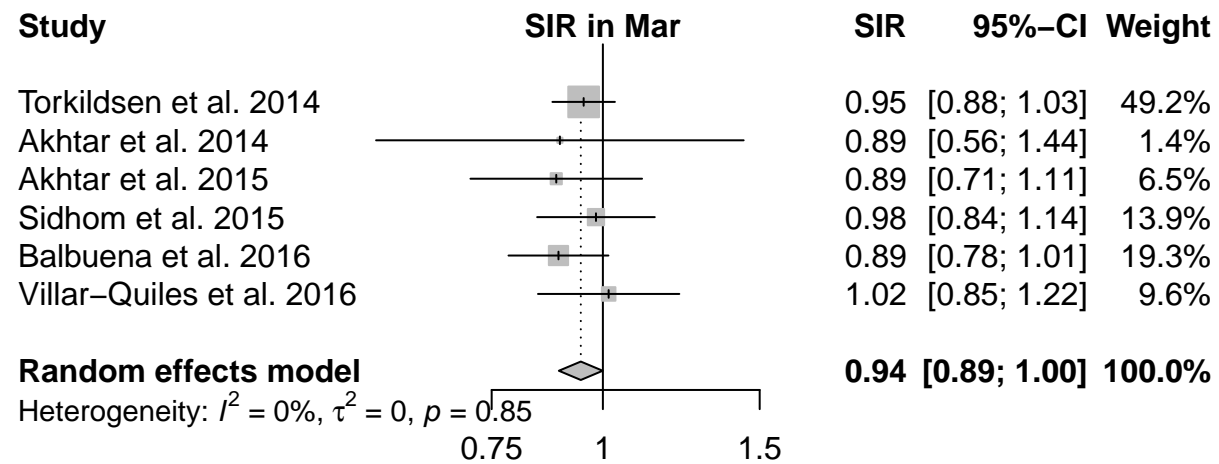

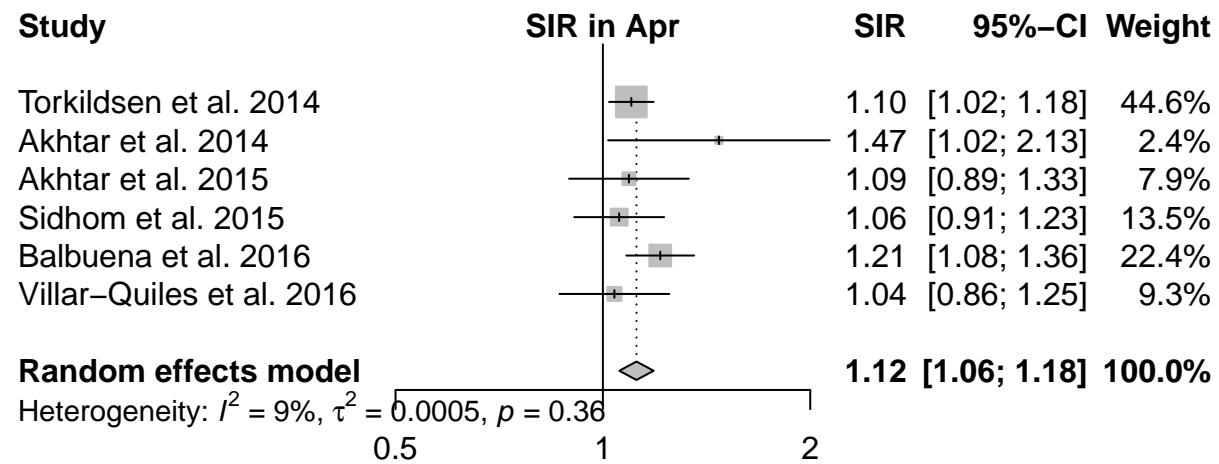

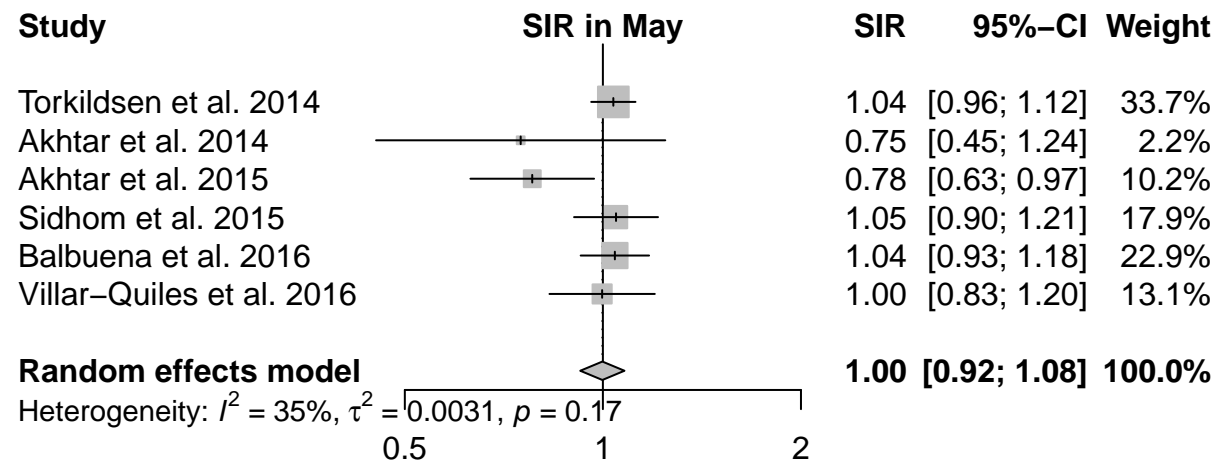

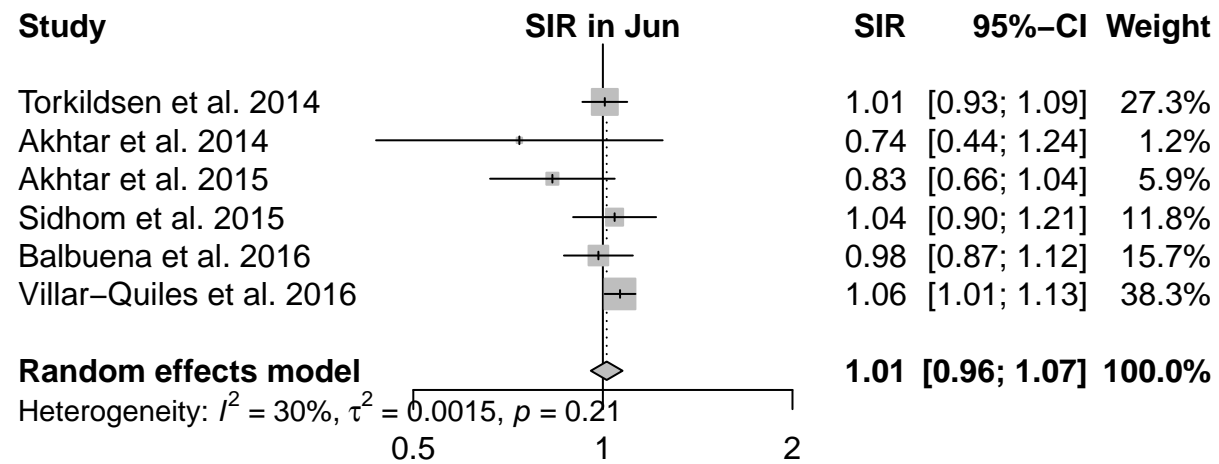

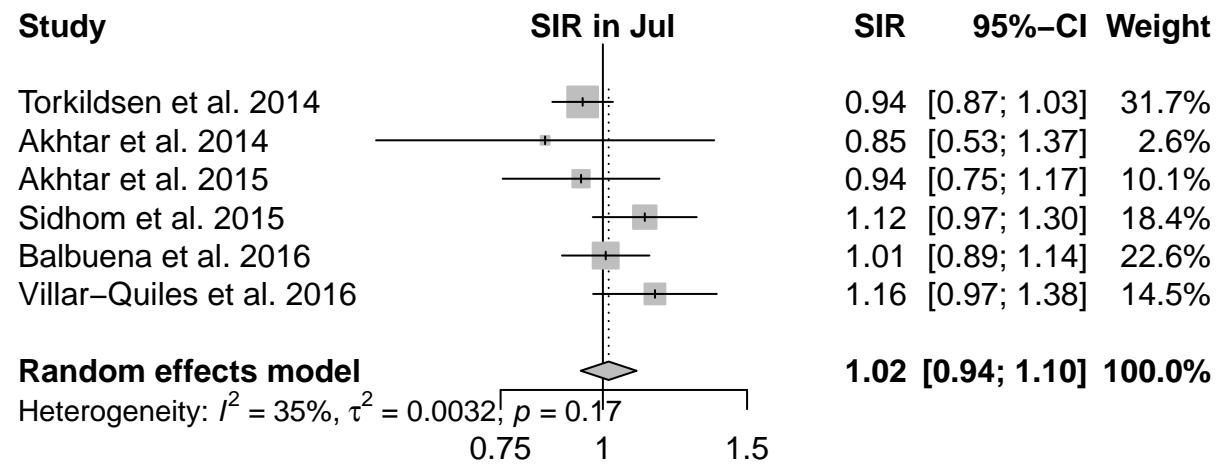

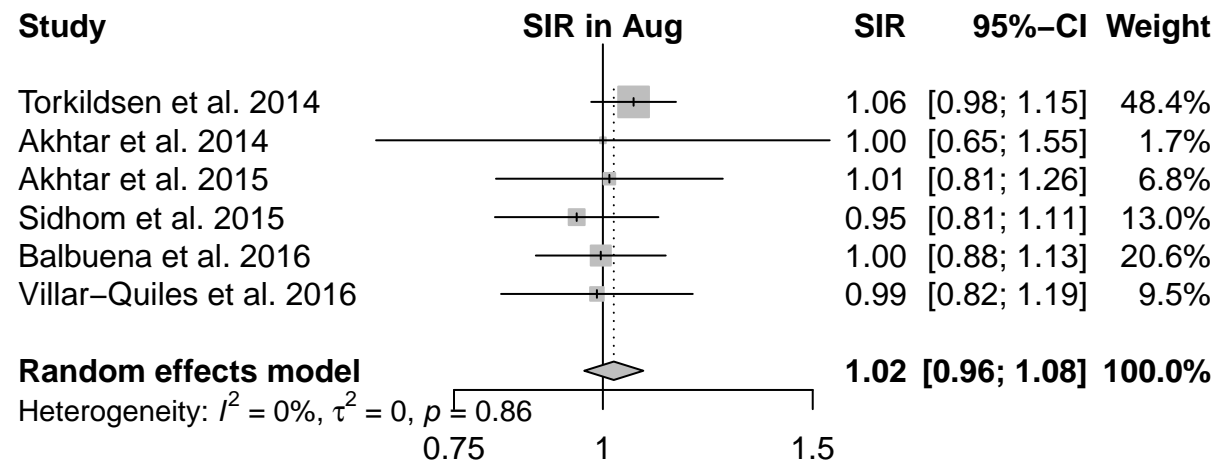

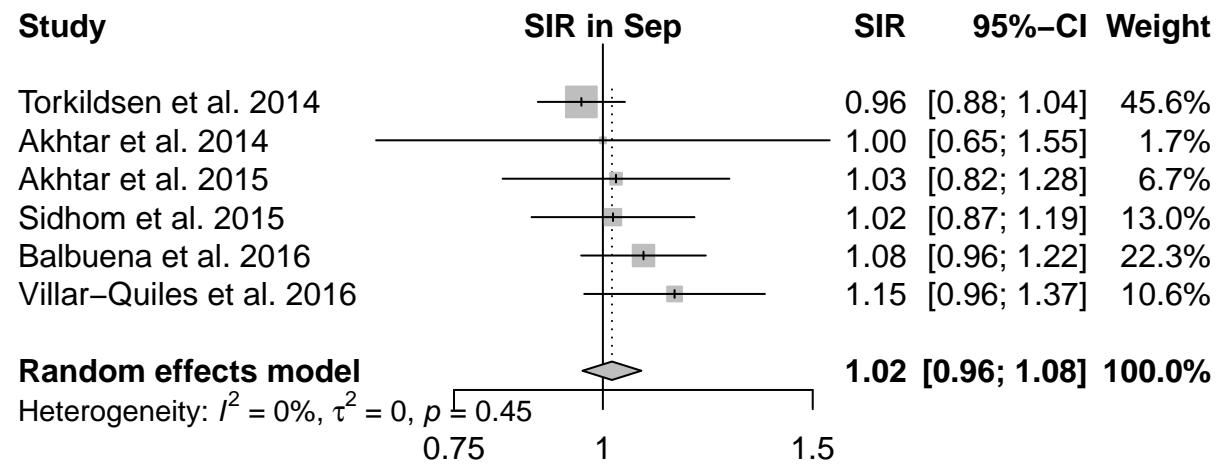

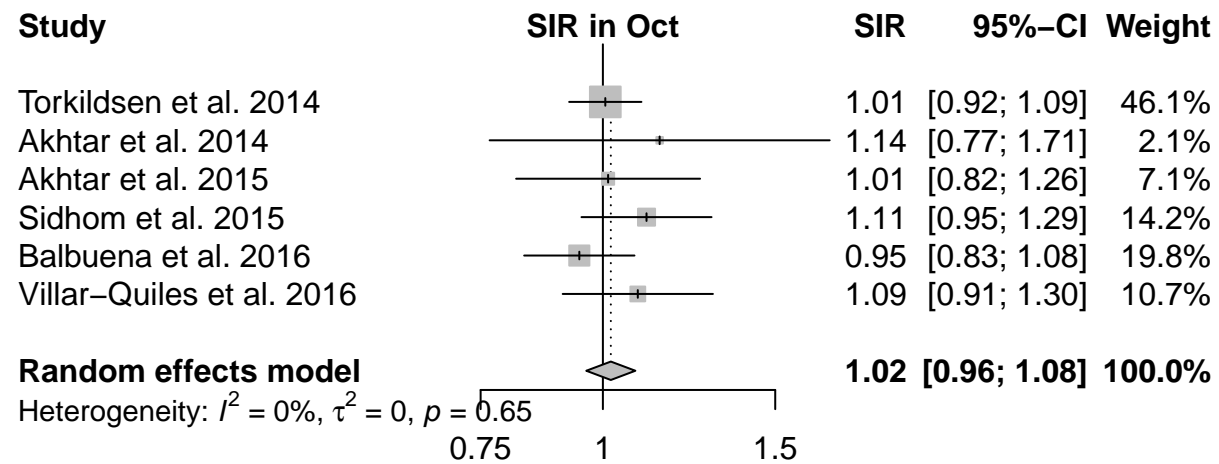

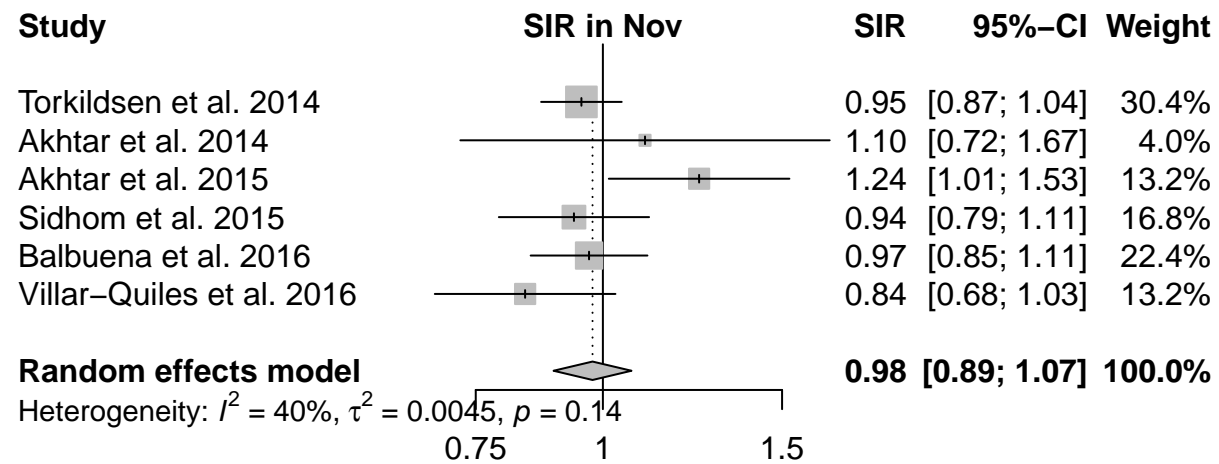

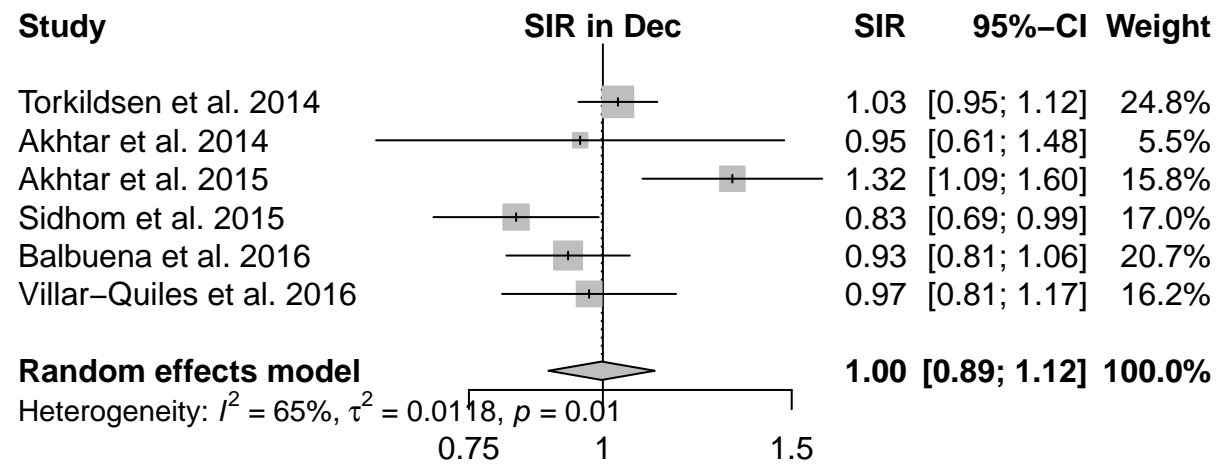

Supplement: S1 Fig — (PDF) [file pone.0221645.s004.pdf]

# Pooled

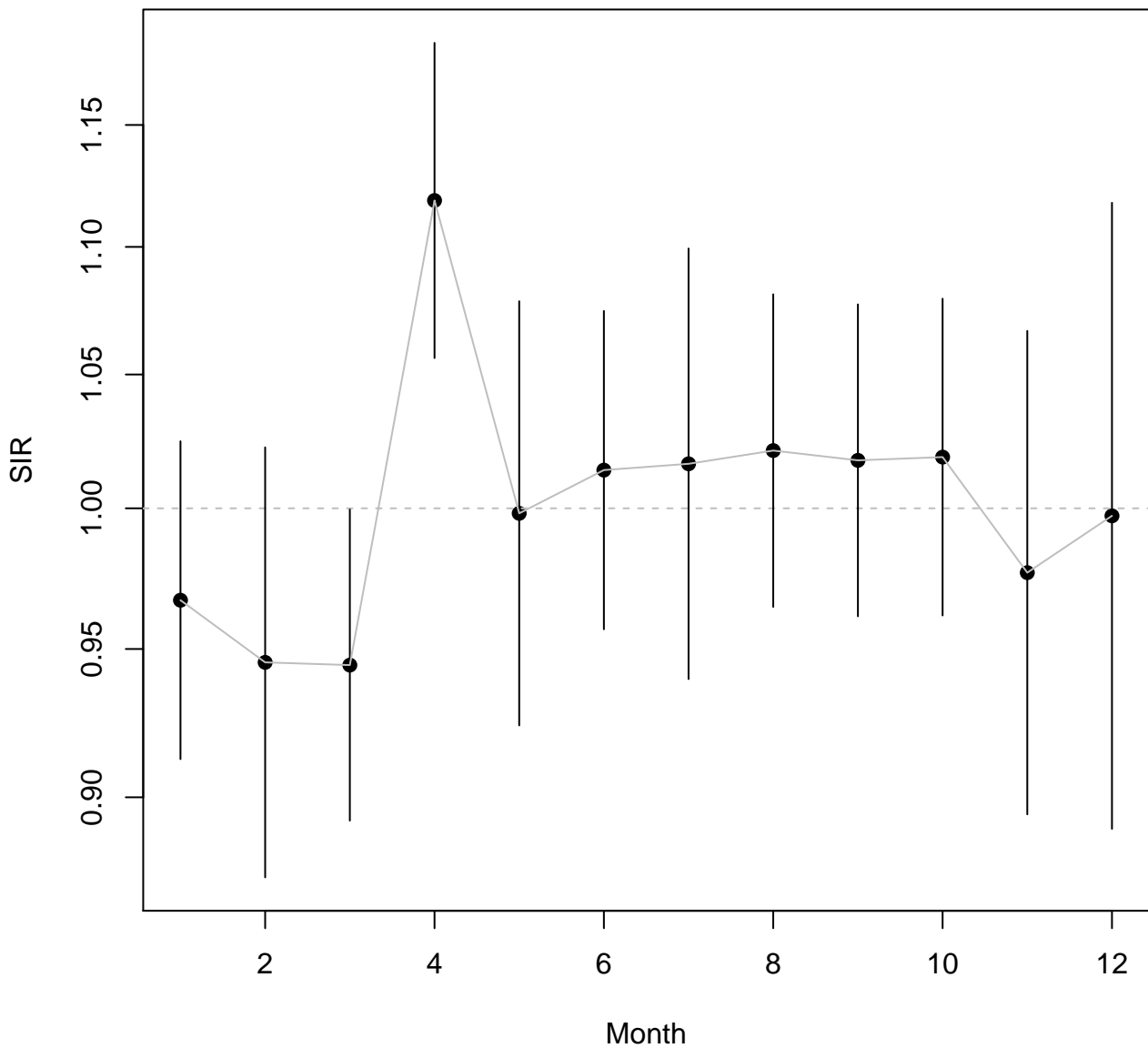

Supplement: S2 Fig — Computed using the weights from the random effects meta-analysis. (PDF) [file pone.0221645.s005.pdf]

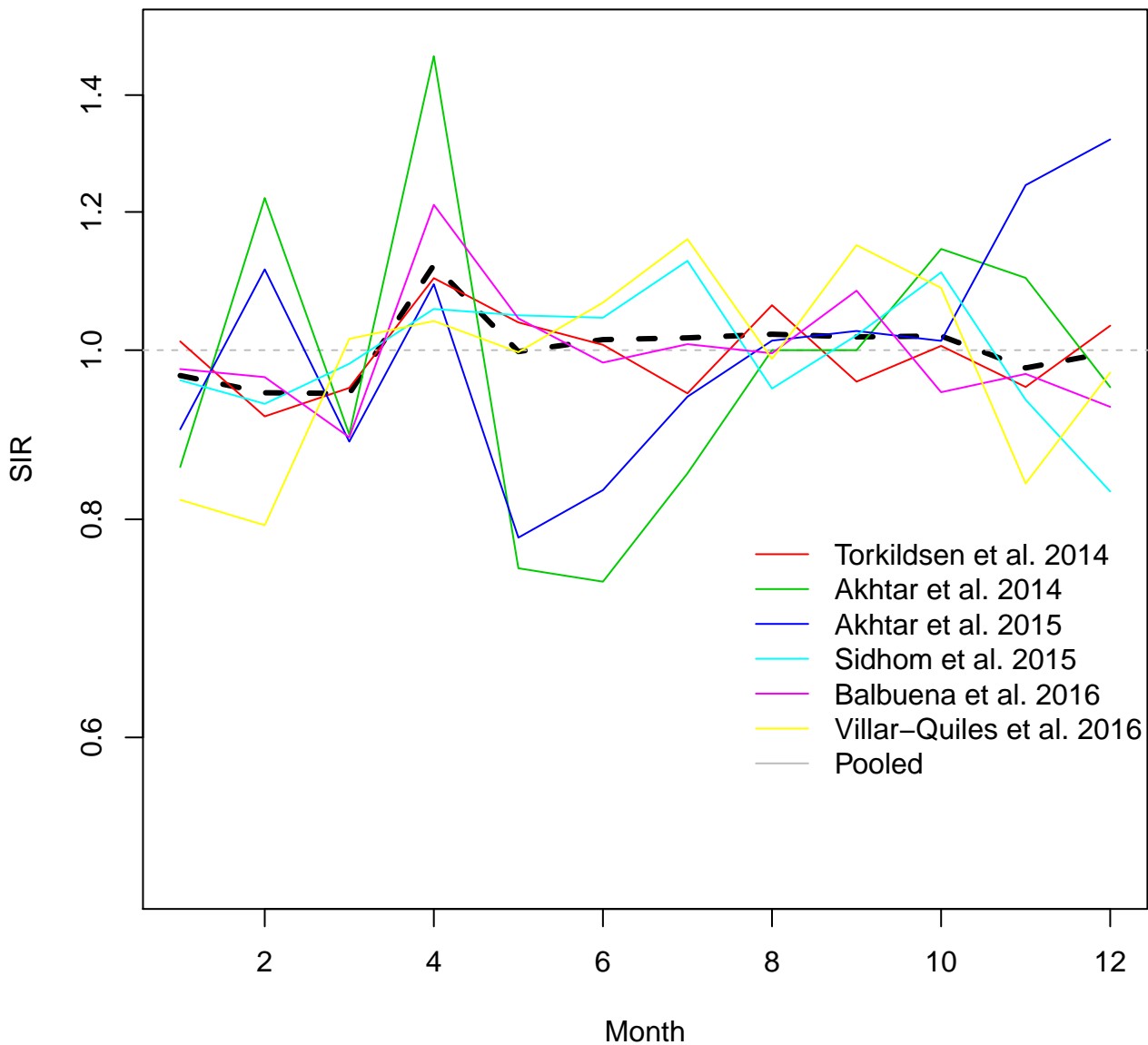

Supplement: S3 Fig — (PDF) [file pone.0221645.s006.pdf]

**Torkildsen et al. 2014**

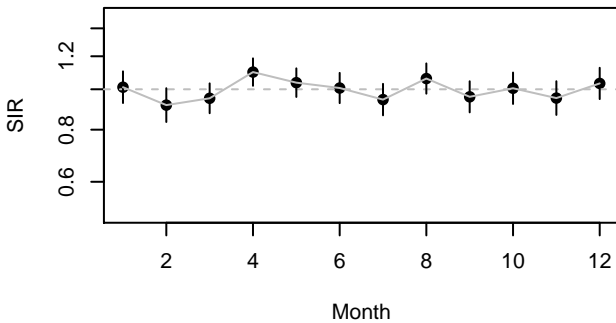

**Akhtar et al. 2014**

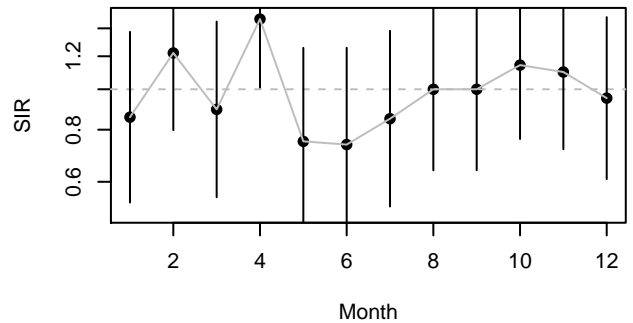

**Akhtar et al. 2015**

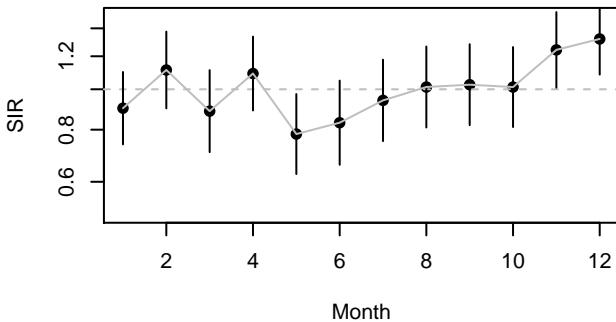

**Sidhom et al. 2015**

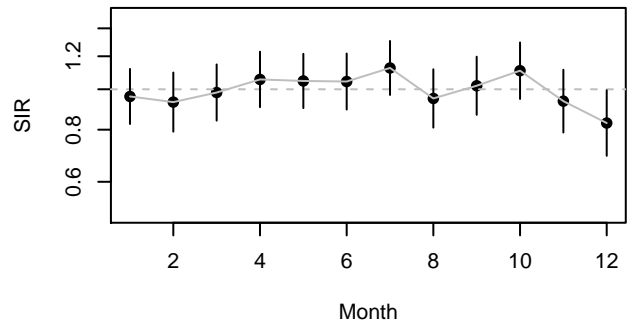

**Balbuena et al. 2016**

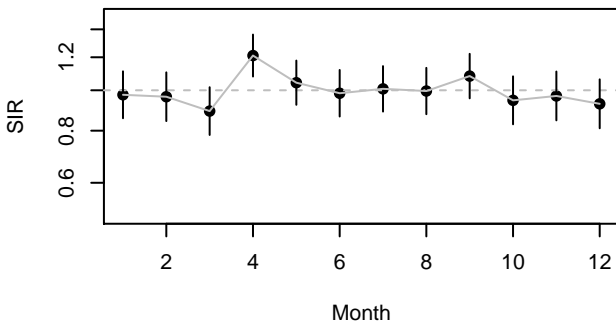

**Villar-Quiles et al. 2016**

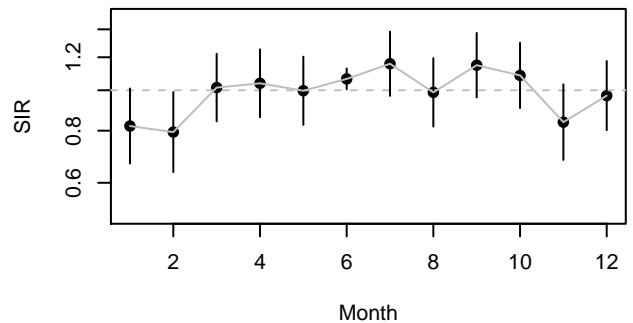

Supplement: S4 Fig — (PDF) [file pone.0221645.s007.pdf]
