# Supplementary material for: Vitamin D in early life and later risk of multiple sclerosis—A systematic review, meta-analysis
Source: PLoS One. 2019 Aug 27;14(8):e0221645. doi: 10.1371/journal.pone.0221645 (PMC6711523; doi:10.1371/journal.pone.0221645)
Supplement: S1 Table — (PDF) [file pone.0221645.s008.pdf]

S1 Table. Search strategy documentation.

<table>
<tr><td rowspan="6">OR</td><th>Population</th><th>Intervention</th><th>Comparison</th><th>Outcome</th></tr>
<tr><td colspan="4">PubMed/Medline search strings</td></tr>
<tr><td colspan="4">AND</td></tr>
<tr><td rowspan="3">Antenatal
Prenatal
Pregnancy
Neonatal
Offspring
Fetus
Fetal</td><td>Search #1
Vitamin D
Vitamin D2
Vitamin D3
Ergocalciferol
Cholecalciferol
Calcitriol
1,25(OH)2D
25-hydroxyvitamin D</td><td></td><td rowspan="3">Multiple sclerosis
MS</td></tr>
<tr><td>Search #2
Season
Seasonality
Month of birth</td><td></td></tr>
<tr><td>Search #3
Immigration
Emigration</td><td></td></tr>
</table>

*All searches were sorted by relevance and search in [All Fields] using MeSH Terms
