# Supplementary material for: Vitamin D in early life and later risk of multiple sclerosis—A systematic review, meta-analysis
Source: PLoS One. 2019 Aug 27;14(8):e0221645. doi: 10.1371/journal.pone.0221645 (PMC6711523; doi:10.1371/journal.pone.0221645)
Supplement: S2 Table — (PDF) [file pone.0221645.s009.pdf]

S2 Table. Methodological quality of included studies.

S2A Table. Study quality of case-control studies based on the Newcastle-Ottawa scale (The following coding instructions were taken from the Newcastle-Ottawa Scale website (attached in S1 File).

First author, y (ref)	Is the case definition adequate?	Representativeness of the Cases	Selection of Controls	Definition of Controls	Comparability of Cases and Controls on the Basis of the Design or Analysis[2]	Ascertainment of exposure	Same method of ascertainment for cases and controls	Non-Response rate	Total scores[3]
**Population studies**									
Salzer et al. 2012 [23]	★	☆	★	★	★☆	★	★	☆	6
Ueda et al. 2014 [24]	★	★	★	★	★★	★	★	☆	8
Cortese et al. 2015 [27]	★	★	★	★	★☆	☆	★	☆	6
Munger et al. 2016 [25]	★	★	★	★	★★	★	★	☆	8
Nielsen et al. 2017 [26]	★	★	★	★	★★	★	★	☆	8
**Season/month of birth studies**									
Barros et al. 2013 [32]	★	★	★	☆	★★	★	★	☆	7
Fragoso et al. 2013 [39]	★	★	☆	★	★★	★	★	☆	7
Verheul et al. 2013 [44]	★	★	★	☆	☆☆	★	★	☆	5
Torkildsen et al. 2014 [33]	★	★	★	☆	★★	★	★	☆	7
Sidhom et al. 2015 [36]	★	★	★	☆	★☆	★	★	☆	6
Poorolajal et al. 2015 [41]	★	★	☆	★	☆☆	★	★	☆	5
Rodriguez Cruz et al. 2016 [45]	☆	★	★	☆	★★	★	★	☆	6

Study quality of case-control studies (continued)

Balbuena et al. 2016 [43]	★	★	★	☆	☆☆	★	★	☆	5
Villar-Quiles et al. 2016 [44]	★	☆	★	☆	★☆	★	★	☆	6

*Each fulfilled (★) star represents if individual criterion within the section was fulfilled

S2B Table. Study quality of cohort studies

First author, y (ref)	Representativeness of the exposed cohort	Selection of the non exposed cohort	Ascertainment of exposure	Demonstration that outcome of interest was not present at start of study	Comparability of cohorts on the basis of the design or analysis[2]	Assessment of outcome	Was follow-up long enough for outcomes to occur	Adequacy of follow up of cohorts	Total scores[1]
**Population studies**									
Mirzaei et al. 2011 [28]	★	★	☆	★	★★	★	★	★	8
**Season/month of birth studies**									
Gardener et al. 2009 [38]	★	NA	★	★	★★	★	★	☆	7
Streym et al. 2013 [42]	★	NA	★	★	★☆	★	★	☆	6
Akhtar et al. 2014 [34]	★	★	★	NA	★☆	★	NA	NA	5
Akhtar et al. 2015 [35]	★	★	★	NA	★☆	★	NA	NA	5
**Migration studies**									
Hammond et al. 2000 [53]									
Cabre et al. 2005 [55]	★	★	★	NA	★★	★	NA	☆	6
McLeod et al. 2011 [54]									

*Each fulfilled (★) star represents if individual criterion within the section was fulfilled; NA; not applicable.

[1] Thresholds for cohort / case control studies: very good studies: 7 to 8 points; good studies: 5 to 6 points; satisfactory: 4 points; unsatisfactory studies: 0 to 3 points

(Adapted for cross-sectional studies)

First author, y (ref)	Representativeness of the sample	Sample size	Non-respondents	Ascertainment of exposure	Comparability of groups on the basis of the design or analysis[2]	Assessment of outcome	Statistical test	Total scores[3]
**Season/month of birth and migration studies**								
Becker et al. 2013 [31]	★	★	NA	★	☆★	★	★	7
Tolou-Ghamari et al. 2015 [40]	☆	☆	NA	★	☆☆	★	★	3
Berg-Hansen et al. 2015 [56]	★	★	NA	★	★☆	★	★	6

*Each fulfilled (★) star represents if individual criterion within the section was fulfilled; NA; not applicable.

[2] For population studies one star (★☆) was given if a study controls for age/sex while two stars (★★) if any additional relevant factors were controlled (e.g. ethnicity, latitude and season of birth); For season/month of birth studies one star (★☆) was given if a study controls for place/year of birth while two stars (★★) if both factors were controlled; For migration studies one star (★☆) was given if a study controls for age/sex while two stars (★★) if any other relevant factors were controlled

[3] Thresholds for cross-sectional studies: very good studies: 5 points; good studies: 4 points; satisfactory studies: 3 points; unsatisfactory studies: 0 to 2 points

S2C Table. Methodological quality of systematic reviews or meta-analysis using the AMSTAR score

First author, y (ref)	A priori design	Duplicate study selection/extraction	Comprehensive literature search	Status of publication as exclusion criterion	Included and excluded studies provided	Characteristics of included studies provided	Quality assessed	Quality used appropriately	Synthesis methods appropriate	Publication bias assessed	Conflict of interest stated	Rating
Torkildsen et al. 2012 [18]	NR	NR	+	+	NR	+	−	−	−	−	+	Moderate
Dobson et al. 2013 [30]	+	NR	+	−	+	+	−	−	+	+	+	Moderate
Gale et al. 1995 [52]	−	−	+	−	−	+	+	+	NA	−	−	Moderate

NA: not applicable; NR: not reported/can't answer; + = yes; - = no
