# Supplementary material for: Vitamin D in early life and later risk of multiple sclerosis—A systematic review, meta-analysis
Source: PLoS One. 2019 Aug 27;14(8):e0221645. doi: 10.1371/journal.pone.0221645 (PMC6711523; doi:10.1371/journal.pone.0221645)
Supplement: S3 Table — (PDF) [file pone.0221645.s010.pdf]

S3 Table. Confounders (Population studies).

First author, y (ref)	Confounders Considered	Additional Confounders	Matched	Stratified by	Did considering for additional confounders changed the results?	Adjusted for relevant MS risk factors (latitude at birth[1]; ethnicity[2]; smoking[3], MS heredity[4]; sun exposure[5]; Epstein-Barr virus[6])
Mirzaei et al. 2011 [28]	Latitude at birth<br>Ancestry<br>Pack-years of cigarette smoking	Smoking during pregnancy<br>Maternal and Paternal Education level<br>Paternal occupation<br>Mothers prepregnancy BMI<br>Daughters' preschool intake of cod liver oil or Multivitamins<br>Mothers' recreational physical activity		Age in months<br>Calendar year	No	1,2,3
Salzer et al. 2012 [23]			Sex<br>Biobank Sampling date<br>Age			
Ueda et al. 2014 [24]		**In early life:**<br>Month of birth<br>Latitude of birth<br>Breastfeeding<br>**In adult life:**<br>25-hydroxyvitamin D exposure Sun exposure<br>Vitamin D intake from dairy products<br>Fatty fish consumption<br>Smoking<br>Body mass index at 20 years of age<br>**Plus:**<br>Ancestry<br>MS heredity<br>Socioeconomic group	Sex<br>Age<br>Residential area		No	1,2,3,4,5
Cortese et al. 2015 [27]	Age<br>Sex	Smoking before disease onset<br>History of infectious mononucleosis<br>Sun exposure<br>Body shape at age 15<br>Education<br>Consumption of fatty fish			No	3,5,6
Munger et al. 2016 [25]		Sex of the child<br>Gestational age at sample collection<br>Season of sample collection	Region of Birth<br>Date of maternal sample collection<br>Date of mother's birth<br>Date of child's birth		A 20.03 ng/mL increase in maternal 25(OH)D level was associated with a non-statistically significant 48% reduced risk of MS in the offspring (RR 0.52, 95% CI 0.22-1.19)	1
Nielsen et al. 2017 [26]	Sex<br>Age<br>Date of Birth	Parental ethnicity<br>Birthweight<br>Gestational age			No	2
