# Supplementary material for: Vitamin D in early life and later risk of multiple sclerosis—A systematic review, meta-analysis
Source: PLoS One. 2019 Aug 27;14(8):e0221645. doi: 10.1371/journal.pone.0221645 (PMC6711523; doi:10.1371/journal.pone.0221645)
Supplement: S4 Table — (PDF) [file pone.0221645.s011.pdf]

S4 Table. Confounders (The season or the month of birth studies).

First author, y (ref)	Confounders Considered	Matched	Most relevant confounders (place of birth[1]; year of birth[2])
Northern hemisphere			
Gardener et al. 2009 [38]	Age in months<br>Calendar year<br>Latitude at birth<br>Paternal occupation<br>Sibship size<br>Pack-years of smoking in adulthood<br>Quantile of energy-adjusted vitamin D intake	n.a	1,2
Streym et al. 2013 [42]	Year of birth	n.a	2
Barros et al. 2013 [32]	n.a	Same birth time<br>Geographical area	1,2
Torkildsen et al. 2014 [33]	Year of birth<br>Place of birth	n.a	1,2
Akhtar et al. 2014 [34]	Gender<br>Nationality	Time interval	2
Akhtar et al. 2015 [35]	n.a	Time interval	2
Tolou-Ghamari et al. 2015 [40]	n.a	n.a	n.a
Poorolajal et al. 2015 [41]	n.a	Same time interval<br>Same hospital	n.a
Sidhom et al. 2015 [36]	n.a	Year of birth	2
Rodriguez Cruz et al. 2016 [45]	n.a	Time and Region	1,2
Balbuena et al. 2016 [43]	n.a	n.a	n.a
Villar-Quiles et al. 2016 [37]	Year of birth<br>Gender	n.a	2
Southern hemisphere			
Becker et al. 2013 [31]	Ethnicity<br>Latitude	n.a	1
Fragoso et al. 2013 [39]	n.a	Same latitude<br>Similar age<br>Ethinicity<br>Socioeconomic level	1,2
Southern and northern hemisphere			
Verheul et al. 2013 [44]	n.a	n.a	n.a
